# Effect of Intermittent Fasting on Weight Loss in Overweight and Obese Adults: A Systematic Review of Clinical Trials

**DOI:** 10.1002/fsn3.72264

**Published:** 2026-09-15

**Authors:** Hafiz Muhammad Irfan Manzoor, Muhammad Tauseef Sultan, Aneela Hameed, Ahmad Mujtaba Noman, Mahnoor Khalid, Maheen Kazmi, Aiman Sohail, Mohamed Fawzy Ramadan, Eliasse Zongo, Muhammad Abdul Rahim, Fahad Al‐Asmari

**Affiliations:** ^1^ Department of Human Nutrition, Faculty of Food Science and Nutrition Bahauddin Zakariya University Multan Pakistan; ^2^ Department of Animal Food Products Technology, Faculty of Food Science and Nutrition Bahauddin Zakariya University Multan Pakistan; ^3^ Department of Clinical Nutrition, Faculty of Applied Medical Sciences Umm Al‐Qura University Makkah Saudi Arabia; ^4^ Laboratory of Research and Teaching in Animal Health and Biotechnology Université Nazi Boni Bobo‐Dioulasso Burkina Faso; ^5^ Department of Food Science & Nutrition, College of Allied Health Sciences Times University Multan Pakistan; ^6^ Department of Food and Nutrition Sciences, College of Agricultural and Food Sciences King Faisal University Al‐Ahsa Saudi Arabia

**Keywords:** body composition, dietary patterns, intermittent fasting, obesity, time‐restricted eating, weight loss

## Abstract

Intermittent fasting (IF), an alternative calorie restriction strategy, supports weight management and may enhance cardiovascular health, improve glycemic control, and offer potential protective effects against cancer, alongside other possible health benefits. The objective of this systematic review is to study the impact of intermittent fasting on weight loss among overweight and obese adults. The original research articles were searched in four electronic databases: PubMed, Cochrane Library, Google Scholar, and ScienceDirect, with specific time frame restrictions. After going through an organized process of screening and eligibility, 26 out of 2307 studies fitting the inclusion criteria were identified. The established methodological procedure observed in this review adhered to the Preferred Reporting Items for Systematic Reviews and Meta‐Analyses (PRISMA 2020) guidelines to ensure transparency and credibility in the approach. All the included studies demonstrated statistically significant reductions in body weight resulting from intermittent fasting interventions over durations ranging from 3 to 50 weeks. However, several trials exhibited comparable effectiveness between intermittent fasting and continuous calorie restriction. The findings suggest that intermittent fasting is an effective approach for weight loss, contributing to favorable changes in body composition, including a decrease in fat mass, an improvement in lean mass, and a reduction in waist circumference. These outcomes collectively contribute to improvements in body mass index (BMI) and overall metabolic health. The risk of bias was assessed using the Cochrane Risk of Bias 2.0 (RoB2) tool for 24 RCT studies, resulting in 16 studies with a low risk of bias, 1 study with a high risk, and 7 studies showing a moderate risk. While two included non‐randomized controlled studies were assessed using the MMAT tool. Different forms of intermittent fasting including TRE (16:8, 20:4, 14:10, and 18:6), Alternate Day Fasting (ADF), and IF (5:2) appear promising for sustainable weight management. Among these, ADF and TRE (particularly 16:8 protocols) demonstrated the most consistent evidence of weight reduction across included studies, with no evidence of statistically significant differences in comparison with continuous calorie restriction. Moreover, future studies should compare different protocols, such as time‐restricted eating and alternate‐day fasting, while exploring underlying physiological mechanisms to guide healthcare professionals in creating effective, personalized dietary strategies for managing overweight and obesity.

## Graphical abstract

This systematic review screened 2307 records identified through PubMed, Cochrane Library, Google Scholar, and ScienceDirect, following PRISMA 2020 criteria. The search yielded 26 eligible clinical trials (24 RCTs, 2 non‐randomized studies) on intermittent fasting (TRE 16:8/20:4/14:10/18:6, alternate‐day fasting, and 5:2 fasting) for weight loss in overweight and obese adults. Risk of bias was assessed using RoB2 (16 low, 7 moderate, 1 high risk) and the MMAT tool for non‐randomized studies. All included studies showed statistically significant reductions in body weight over 3–50 weeks, with improvements in fat mass, lean mass, and waist circumference. Several trials found intermittent fasting comparable in effectiveness to continuous calorie restriction in reducing weight, with ADF and TRE (16:8) protocols showing the most consistent evidence of weight loss.

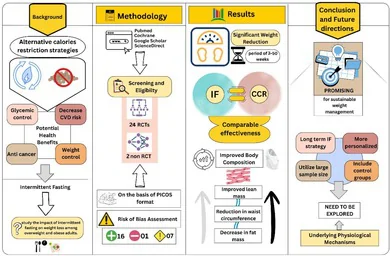

## 1 Introduction

Obesity and overweight have emerged as major global health challenges, particularly among individuals leading sedentary lifestyles and consuming energy‐dense, highly processed, and nutritionally poor diets. According to the World Health Organization (2020), the global prevalence of overweight and obesity has tripled since 1975. By 2022, over 2.5 billion adults aged 18 and above were classified as overweight, with approximately 890 million categorized as obese. These statistics underscore the urgent need for effective dietary strategies to manage and mitigate the rising burden of excess weight. Several studies have demonstrated that extending overnight fasting durations can significantly improve overall health and contribute to weight reduction (Erdem et al. 2022).

Nutritional interventions and physical activity programs designed to restore energy balance, either by reducing caloric intake or increasing energy expenditure, have demonstrated positive outcomes in weight management efforts. Although traditional approaches are well‐established, emerging evidence highlights intermittent fasting (IF) and high‐intensity interval training (HIIT) as particularly effective strategies for sustainable weight loss (Wilson et al. 2018). While numerous dietary interventions exist for weight loss, intermittent fasting has garnered increasing scientific and clinical recognition over the past several decades (Klempel et al. 2012). Short‐term clinical trials indicate that IF can effectively promote weight loss by reducing fat mass in overweight and obese populations (Wilson et al. 2018). Intermittent fasting refers to a voluntary abstention from food and/or caloric beverages for defined periods. Historically, fasting practices were observed globally across various cultures and traditions in diverse formats (Patterson and Sears 2017).

Intermittent fasting has gained popularity as a straightforward dietary strategy, characterized by structured eating windows interspersed with defined fasting periods, depending on the protocol adopted (Lowe et al. 2020). Common IF protocols include:

- Alternate Day Fasting (ADF): Alternating between fasting or calorie‐restricted days and days of unrestricted eating.
- Warrior Diet: Consuming raw fruits and vegetables during the day, followed by a single large meal at night after a ~20‐h fast.
- 5:2 Diet: 5 days of normal eating combined with 2 days (not consecutive) of eating 500–600 kcal.
- 16:8 Protocol: 16 h of fasting and 8 h of feeding every day.
- 18:6 Process: 18‐h fasted state and a 6‐h feeding window.

The practice of fasting is deeply rooted in religious and cultural beliefs and takes many different forms around the world (Salis et al. 2022). Recent reviews have demonstrated that fasting induces cellular mechanisms that enhance the body's antioxidative protection and mitigate metabolic‐related stress. It results in the better repair of damaged biomolecules, the regulation of metabolic homeostasis, and ultimately, aids in weight loss (Diab et al. 2024; Song and Kim 2023; Martínez‐Rodríguez et al. 2021). Alternate Day Fasting (ADF) has been shown to lead to remarkable results in terms of manipulating hunger and satiety signals, particularly in obese adults (Catenacci et al. 2016). Research on Ramadan fasting and body weight has provided conflicting evidence in its findings, with some studies reporting significant decreases in body weight and others showing little to no effect (Ahmed et al. 2021).

Several systematic reviews have provided updated and in‐depth knowledge on different types of intermittent fasting while conducting meta‐analysis and covering parameters other than weight loss (Semnani‐Azad et al. 2025; Sun et al. 2024; Cho et al. 2019; Harris et al. 2018). However, our manuscript contains several important points to consider. This systematic review aims to critically assess the impact of intermittent fasting on overweight and obesity by examining the growing body of literature spanning from 2000 to 2025, with the goal of informing evidence‐based interventions for managing the obesity epidemic. Our systematic review clearly states its protocol in order to address protocol heterogeneity, keeping in view any confounding factors like use of weight loss medications; such articles are excluded. It focuses on the most commonly practiced forms of intermittent fasting and provides updated data on its types instead of covering a wide range of types, which may otherwise make results less specific. Furthermore, this review has covered the overweight or obese population but may also have comorbid conditions. Exercise combined interventions may increase the efficacy of the intervention; therefore, such types of intermittent fasting are included in the scope of this review. In addition to this, different nature of data can be gained through different study types, so both RCTs and non RCTs are evaluated for results. Overall, the present study evaluates the effectiveness of intermittent fasting and its various protocols in promoting weight loss among overweight and obese adults.

## 2 Methodology

A comprehensive and systematic search was conducted from April 10 to April 15, 2026, using four electronic databases: PubMed, Google Scholar, ScienceDirect, and Cochrane Library, for original research articles. A total of 2307 studies were retrieved (310 from PubMed, 995 from Google Scholar, 518 from Cochrane, and 484 from ScienceDirect). Articles published between 1st January 2000 and 15th July 2025 were considered.

### 2.1 Protocol

Three reviewers independently assessed the studies for inclusion, resolving any discrepancies through consensus. The selection process followed the PICOS framework (Population, Intervention, Comparison, Outcome, and Study Design), with the criteria detailed below:

- Population: Adults of both genders aged 18–75 years, representing early and middle adulthood, with a BMI ≥ 25 kg/m^2^ or diseased adults who are overweight or obese.
- Intervention: Studies implementing any form of intermittent fasting, including but not limited to ADF, 16:8 TRF, 4‐h or 6‐h TRF, and 5:2 fasting (involving 2 days of fasting, either consecutive or non‐consecutive). Interventions combined with exercise (e.g., resistance training, high‐intensity workouts) or specific nutrient‐restriction strategies (e.g., protein pacing, low‐carbohydrate or high‐fat diets) were also included.
- Comparison: Only studies comparing intermittent fasting interventions with a group declared as control group were included. Control groups consisted of participants following ad libitum diets, normal diets, habitual diets, calorie restricted diets, or performing exercise combined with those mentioned previously. Studies comparing two forms of intermittent fasting (IF) with another type of diet not declared as control were excluded.
- Outcomes: Studies were included if they reported anthropometric or body composition indicators such as body weight, BMI, waist circumference, total or percentage body fat, lean mass, visceral fat, or hip circumference.
- Study Design: Excluded publications included preclinical trials, observational studies, protocols, reviews, meta‐analyses, theses, books, preliminary studies, short reports, pilot and observational studies, preprints, clinical cases, editorials, opinion pieces, case reports, and gray literature. Only human subject studies were included. Participants using weight‐loss or weight‐gain medications, or medications that may exert a positive or negative effect on weight outcomes, were excluded to avoid confounding. Studies with durations of at least 15 days were considered.

### 2.2 Search Strategy

The search strategy employed a combination of Medical Subject Headings (MeSH) and free‐text terms including: “intermittent fasting” OR “intermittent fasting 16 8” OR “Ramadan fasting” OR “intermittent fasting 5 2” OR “intermittent fasting diet” OR “intermittent fasting regimens” OR “intermittent fasting approach” OR “intermittent fasting interventions” OR “Alternate day fasting” OR “time‐restricted eating” OR “time‐restricted feeding” OR “intermittent energy restriction” AND “overweight” OR “obese” OR “weight loss” AND “adults” OR “patients” OR “humans.” PubMed searches were refined using filters for Clinical Study, Clinical Trial, Controlled Clinical Trial, Randomized Controlled Trial, and an age restriction of 18–75 years. Moreover, the search was conducted in Cochrane Library using filters including study type (trial) and date published on Cochrane Library (2000–2025). Google Scholar was used as a supplementary search source to identify additional potentially relevant studies not captured in database searches. The use of Google Scholar enhances the sensitivity of search strategy by minimizing the risk of missing eligible studies. In order to prevent exclusion of eligible studies where protocol terms are not present in the title, the search was conducted broadly without restricting retrieval to title words only. A custom year filter (2000–2025) was applied. The search yielded 19,300 records. Because Google Scholar provides access to only the first 100 pages of results, a structured and reproducible screening approach was implemented in which results were screened in the order presented by Google Scholar (sorted by relevance). All 995 records available within this limit were screened. Titles together with available abstracts were assessed rather than titles alone to minimize the risk of excluding potentially eligible studies. To further enhance coverage and compensate for limitations of Google Scholar, an additional structured database (ScienceDirect) was searched using predefined search terms with filters applied such as custom year (2000–2025), and research articles only. Consequently, 26 studies were selected using the inclusion and exclusion criteria from four databases including PubMed, ScienceDirect, Google Scholar, and Cochrane Library for this review adhering to the 2020 PRISMA guidelines. The complete search strategy for all the databases including PubMed, Cochrane Library, Google Scholar, and ScienceDirect is provided in Table 1.

**TABLE 1:** Search strategies for all databases.

Database	Date of search	Boolean search string	Filters/Limits applied
PubMed	April 10, 2026	((intermittent fasting [MeSH Terms]) OR (intermittent fasting 16 8) OR (Ramadan fasting) OR (intermittent fasting 5 2) OR (intermittent fasting diet) OR (intermittent fasting regimens) OR (intermittent fasting approach) OR (intermittent fasting interventions) OR (Alternate day fasting) OR (time‐restricted eating) OR (time‐restricted feeding) OR (intermittent energy restriction)) AND ((overweight [MeSH Terms]) OR (obese) OR (weight loss)) AND ((adults[MeSH Terms]) OR (humans [MeSH Terms]) OR (patients))	Article type: Clinical study, Clinical Trial, Controlled Clinical Trial, Randomized Controlled Trial, Article language: English, Species: Humans, Sex: Female and Male, age: Adult: 19+ years, Young Adult: 19–24 years, Adult: 19–44 years, Middle Aged + Aged: 45 + years, Middle Aged: 45–64 years, Aged: 65+ years. Exclude Preprints, custom year range: 2000/1/1 to 2025/7/15
Cochrane Library	April 11, 2026	#1 MeSH descriptor: [intermittent fasting] #2 ((“intermittent fasting” OR “intermittent fasting 16 8” OR “Ramadan fasting” OR “intermittent fasting 5 2” OR “intermittent fasting diet” OR “intermittent fasting regimens” OR “intermittent fasting approach” OR “intermittent fasting interventions” OR “Alternate day fasting” OR “time‐restricted eating” OR “time‐restricted feeding” OR “intermittent energy restriction”)): ti, ab, kw #3 (#1 OR #2) #4 MeSH descriptor: [Overweight] #5 MeSH descriptor: [Weight loss] #6 (Overweight OR Obese OR weight loss): ti, ab, kw #7 (#4 OR #5 OR #6) #8 MeSH descriptor: [Adult] #9 MeSH descriptor: [Patients] #10 (Adults OR Patients OR Humans): ti, ab, kw #11 (#8 OR #9 OR #10) #12 (#3 AND #7 AND #11)	Content type: Trials, Custom range: 01‐01‐2000 to 15‐07‐2025
Google Scholar	April 12–14, 2026	(“intermittent fasting” OR “intermittent fasting 16 8” OR “Ramadan fasting” OR “intermittent fasting 5 2” OR “intermittent fasting diet” OR “intermittent fasting regimens” OR “intermittent fasting approach” OR “intermittent fasting interventions” OR “Alternate day fasting” OR “time‐restricted eating” OR “time‐restricted feeding” OR “intermittent energy restriction”) AND (“overweight” OR “obese” OR “weight loss”) AND (“adults” OR “patients” OR “humans”)	Custom year range: 2000–2025
ScienceDirect	April 15, 2026	(“intermittent fasting” OR “time‐restricted eating” OR “alternate day fasting” OR “intermittent energy restriction”) AND (“weight loss” OR “body composition”) AND (overweight OR obese) AND (adults)	Year range: 2000–2025, and article type: Research articles

### 2.3 Eligibility Criteria

#### 2.3.1 Inclusion Criteria

The inclusion criteria were set in accordance with the PICOS format as follows: (i) Population: Studies involving only human subjects were included; these studies consisted of both genders (males and females) and the age group specified was 18–75 years, must be overweight or obese, (ii) Intervention and Comparators: Studies including any form of intermittent fasting, such that it might be modified in terms of calories, nutrients or hours division, and compared with a control group following usual diet or calorie restriction, (iii) Outcomes measured: Studies included if they measured body composition or anthropometric measures, (iv) Study design: Controlled clinical studies dealing with humans only were included for analysis.

#### 2.3.2 Exclusion Criteria

The studies that were excluded from systematic review were as follows: (i) The studies that were conducted in vitro or in vivo (ii) Publications including preclinical trials, observational studies, protocols, reviews, meta‐analyses, editorials, opinion pieces, case reports, and gray literature (iii) Studies not involving weight or anthropometrics or body composition measures as outcomes or if at least any one of these not present with other types of measures in the study (iv) Studies published in language other than English (v) Studies that had a trial period less than 15 days (vi) Studies involving any confounding factors that may affect (enhance or underestimate) the measurement or magnitude of the ultimate desirable outcome.

### 2.4 Literature Screening

Following the removal of duplicates, the combined results were screened by three reviewers. Titles and abstracts were assessed to exclude studies based on criteria such as duplication, irrelevant interventions, non‐relevant outcomes, an ineligible population (due to age or BMI), or the absence of a control group. Full‐text reviews were then conducted for the remaining studies. Articles were excluded if they used medications such as insulin, metformin, or relied solely on meal replacements. Ultimately, 26 studies were included: 24 were randomized controlled trials (RCTs) and 2 were non‐randomized trials.

### 2.5 Data Extraction Process

Three researchers independently conducted the data extraction process on printed forms, and the results were subsequently cross‐verified to ensure transparency, consistency, and accuracy in the extracted information. A structured extraction template was developed to systematically retrieve relevant information from the selected studies. Utilizing this template, the extracted data encompassed baseline characteristics such as participants' age, gender, weight, BMI, and existing comorbidities, along with publication details (journal name, authors, year of publication), study design, duration, sample size, and specifications of intervention and control groups, as shown in Table 2.

**TABLE 2:** Characteristics of included studies (*n* = 26).

Study	Sample size (*n*)^a^	Study design^b^	Duration	Intervention	Intervention detail	Control group	Reported body weight or mass or BMI^c^ (kg or % or kg/m^2^)	Comparison with control group	Comparison between intervention groups (if any)
Oldenburg et al. (2025)	81 (completed)	Participants with age group: 18–65 years, BMI: 30–55 kg/m^2^, 48 females and 40 males (enrolled)	12 weeks	8 h TRE	The subjects fasted for 16 h, with 8 h eating window in a day (for those subjects having difficulty in following, they followed 10 h eating, and 14 h fasting window, for first 1–2 weeks and then moved to 8 h eating window)	Unrestricted eating (UE)	−3.0 kg weight in TRE group, −1.6 kg weight in UE group	TRE group resulted in greater weight reductions than UE group (difference: −1.4 kg)	There were no significant differences between TRE and CR group (mean weight difference: 1.1 kg)
				Calorie reduction group (CR: 15%)	The group participants followed 15% reductions in calorie intake below weight maintenance		−4.1 kg weight in CR group, −1.6 kg in UE group	CR group participants demonstrated greater weight loss than UE group	
Shafiee et al. (2025)	42	Overweight and obese patients with MAFLD, age group: > 18 years, BMI: ≥ 25 kg/m^2^, 26 males and 16 females	12 weeks	TRF (16:8) and lacto‐ovo‐vegetarian (LOV) diet	The group participants fasted for 16 h, followed by 8 h eating window and lacto‐ovo‐vegetarian diet was consumed	Usual diet	−8.07 ± 4.31 kg (8.9% weight loss) in TRE + LOV group, −3.75 ± 3.97 kg (4.2% weight loss) in control group	The intervention group depicted notable weight loss than the control group	Not applied
Tsitsou et al. (2025)	59	MASLD patients with overweight or obesity, age: > 18 years, BMI: ≥ 25 kg/m^2^, 27 males and 32 females	20 weeks (12 weeks intervention period and follow‐up at 20 weeks)	Early TRE (eTRE; 14:10) based on Mediterranean diet principles	In eTRE group, the participants had eating window of 10 h (8 a.m–6 p.m.), and fasted for remaining 14 h, following hypo chloric diet in eating window (500 kcal reductions in resting energy expenditure) for 12 weeks	Hypocaloric mediterranean diet (500 kcal energy restriction with CHO: 45%, protein: 20% and fat: 35%)	TRE group (baseline: 88.2 ± 3.4 to 80.7 ± 3.3 kg weight at the end of 20 weeks), Control group (baseline: 93.9 ± 3.7 to 85.0 ± 36 kg weight [end of 20 weeks])	No significant difference between eTRE and control group reported	At the end of follow‐up period (20 weeks), there found no differences between the intervention groups
				Late TRE (lTRE; 14:10) based on Mediterranean diet principles	14 h fasting (zero calorie beverages allowed), with 10 h eating (12 p.m.–10 a.m.), and following a hypocaloric diet (500 kcal below resting energy expenditure)		eTRE group (baseline: 96.2 ± 3.7–88.9 ± 3.6 kg weight at the end of 20 weeks), control group (baseline: 93.9 ± 3.7–85.0 ± 36 kg weight at end of 20 weeks)	The study found no difference between the intervention and control group	
Martínez‐Montoro et al. (2025)	140 (completed)	Obese participants, age: 18–65 years, BMI: 30–45 kg/m^2^, 47 males and 113 females (enrolled)	3 months	Ketogenic diet (KD)	The KD group followed a very low carbohydrate diet (CHO: 5%, protein: 30% and fat: 65%), with 600 kcal energy deficit	Mediterranean diet (MedDiet: plant‐based diet following CHO: 45%, protein: 20% and fat: 35%, with 600 kcal deficit)	KD: −11.91 kg weight, MedDiet: −8.41 kg weight	KD group reported significant weight loss than control group (difference: −3.78 kg)	mADF and KD groups found significant weight loss than other intervention groups
				Early TRE (eTRE)	eTRE group followed an 8 h eating window (8 a.m.–4 p.m.) with CHO: 45%, protein: 20% and fat: 35% and 600 kcal energy restriction, followed by 16 h fasting		eTRE: −9.43 kg weight, MedDiet: −8.41 kg weight	eTRE group didn't show significant differences than control (difference: −1.22 kg)	
				Late TRE (lTRE)	lTRE consumed 45% CHO, 20% protein and 45% fat, with 600 cal restriction in an 8 h eating window (2–10 p.m.), followed by 16 h fasting		lTRE: −10.56 kg weight, MedDiet: −8.41 kg weight	lTRE participants demonstrated significant weight loss than control (difference: −2.27 kg)	
				Modified alternate day fasting (mADF)	mADF group was subjected to 24 h normal caloric intake (CHO: 45%, protein: 20% and fat: 35%), followed by modified 24 h (25%–30% of the calorie requirements, with 400–800 kcal 3 days a week)		mADF: −11.80 kg weight, MedDiet: −8.41 kg weight	mADF group observed significant weight loss in comparison with control group (difference: −3.14 kg)	
Talebi et al. (2024)	90 (dropout not mentioned)	Obese women with PCOS, age group: 18–40 years, BMI: 25–35 kg/m^2^, 90 females and 0 males	8 weeks	14:10 early TRE (eTRE) + probiotic supplementation	The subjects consumed ad libitum meals for 10 h (8 a.m.–6 p.m.), followed by 14 h fasting and probiotic capsules ( *Lactobacillus rhamnosus* and *Lactobacillus reuteri* , to be consumed with a glass of water after breakfast)	Daily calorie restriction (DCR: 1500–1800 kcal with three meals and snacks a day), and a placebo supplement (130 mg starch), to be consumed with water after breakfast	−2.2 ± 1.6 kg weight in eTRE+probiotic group, −2.5 ± 1.7 kg weight in DCR group	No differences in weight loss between eTRE + probiotic supplementation and DCR with placebo supplementation group	There were no significant differences in results between both intervention groups
				14:10 eTRE+ placebo supplement	14 h fasting window, followed by 10 our eating without any calorie restriction, and placebo (130 mg starch) supplement		−2.9 ± 2.7 kg weight in eTRE+placebo group, −2.5 ± 1.7 kg weight in DCR group	Mean weight loss was similar between eTRE + placebo and DCR with placebo supplementation groups	
Quist et al. (2024)	92 (completed)	Overweight and obese adults with or without prediabetes, age: 30–70 years, BMI: ≥ 25 kg/m^2^, 66 females and 34 males (enrolled)	6 months (3 months intervention and 3 months follow up)	TRE (10 h)	The participants consumed all foods and drinks in self‐selected 10 h eating period (starting from 2 h after wakeup to end at 3 h before bedtime) for 13 weeks	Habitual living	TRE: −1.1 kg weight Control: −0.9 kg weight	There were no significant differences in weight between intervention and control group	Not applied
Rizvi et al. (2024)	90	Participants of age group: 40–60 years, BMI: > 25 kg/m^2^, 56 males and 34 females	12 weeks	IF (16:8)	16 h fasting (9–1 p.m.) followed by 8 h eating window	Normal diet (ND)	IF 16:8 (ΔBMI: 10.04% ± 4.15%), ND (ΔBMI: 0.47% ± 1.08%)	No significant change in BMI observed between IF and ND groups	There were no significant differences in BMI between both intervention groups
				Customized diet (CD)	Subjects received the diet plans according to their BMI by certified dietitians		CD: (ΔBMI: 1.25% ± 1.08%), ND (ΔBMI: 0.47% ± 1.08%)	The differences in BMI between CD and ND groups were not statistically significant	
Domaszewski et al. (2023)	108	Older people with age: 65–74 years, BMI: 25–29.9 kg/m^2^, 57 females and 51 males	6 weeks	Time‐restricted eating (TRE; 16:8)	The group subjects fasted for 16 h (8 p.m.–12 .a.m next day), followed by 8 h eating	Diet plan according to previous eating habits	TRE: −1.8 kg weight in males and −1.3 kg weight in females; Control: +0.7 kg weight in males and −0.5 kg weight in females	TRE group demonstrated better results than control group	Not applied
Hooshiar et al. (2023)	47	Overweight or obese women, age: 18–50 years, BMI: 25–40 kg/m^2^, all females	8 weeks	Alternate day modified fasting (ADMF)	Intermittent fasting and feeding periods every other day (25% energy deficit on fasting days and 100% of the energy needs consumption on feast days)	Calorie restriction (CR: 63% of the calculated calories, with 3 meals and snacks a day and CHO: 55%, protein: 15% and fat: 30%)	−5.23 kg weight in ADMF group, −3.15 kg weight in control group	ICR group demonstrated greater weight loss than the control group	Not applied
Lin et al. (2023)	77 (completed)	Obese adults, age group: 18–65 years, BMI: 30–50 kg/m^2^, 74 females and 16 males (enrolled)	12 months (6 months weight loss and 6 months weight maintenance phase)	TRE (16:8)	In the weight loss phase, the subjects followed a 16‐h fasting with an 8‐h eating window (ad libitum meals from noon to 8 p.m., and fasting from 8 p.m. to noon) In weight maintenance phase, 14 h fasting (8 p.m.–10 a.m.) and 10 h eating window (10 a.m.– p.m.) was followed	Normal eating habits (> 10 h a day)	−3.49 kg weight in TRE group, +1.12 kg weight in control group	TRE group showed greater weight loss than control group (mean difference: −4.61 kg)	No significant difference between TRE and CR groups were reported (mean difference: 0.81 kg)
				Calorie restriction (CR: 25%)	In weight loss phase, 25% reduction in calories intake while in weight maintenance phase, 100% of the energy needs were consumed.		−4.30 kg in CR group, +1.12 kg in control group	CR group revealed significant reductions in body weight than control (mean difference: −5.42 kg)	
Ezpeleta et al. (2023)	74	Adults with obesity and NAFLD, age 23–65 years, BMI range: 30–60 kg/m^2^, 61 females and 13 males	3 months	ADF with aerobic exercise	Consumed 600 kcal as a dinner (between 5:00 p.m. and 8:00 p.m.) on fast days and participated in a moderate‐intensity aerobic exercise program 5 times per week for 3 months	Control group did not receive any dietary advice	ADF + exercise (−4.58% weight loss), control (−0.60% weight loss)	ADF + exercise group lost more weight than control.	ADF + exercise group reported greater weight loss among all intervention groups however the weight loss did not significantly differ among the ADF and exercise and ADF group alone. Moreover, ADF group showed more weight reductions than exercise group.
				ADF alone	ADF group was instructed to consume 600 kcal as a dinner on fast days and eat food as desired on alternating feast days.		ADF group (−5.06% weight loss), control (−0.60% weight loss)	ADF group lost more weight than control.	
				Exercise alone	Participated are subjected to a moderate‐intensity aerobic exercise program 5 times per week for 3 months		Exercise group (−2.11% weight loss) control (−0.60% weight loss)	Exercise group demonstrated greater weight reductions than control	
Witjaksono et al. (2022)	50	Obese employee, age group: 19–59 years, BMI: > 25 kg/m^2^, 50 males and 0 females	8 weeks	IF (5:2)	In IF (5:2) group, the participants fasted for 2 days (Monday and Thursday for ±14 h a day), followed by ad libitum meals on remaining 5 days of the week	No fasting	−0.8 kg weight in IF (5:2) group, −0.3 kg weight in control group	IF (5:2) participants underwent more weight loss in comparison with control group	Not applied
Chair et al. (2022)	100	Overweight & obese adults with prediabetes, age group: 18–65 years, BMI: ≥ 23 kg/m^2^, 37 males and 64 females (enrolled)	3 weeks	Alternate day fasting (ADF)	In ADF group, 600 kcal on the fasting day with ad libitum on alternating eating day	Usual diet	Numerical value not provided (GEE analysis conducted)	ADF showed better results compared to the control group	ADF demonstrated more significant weight reduction among all study groups
				Time restricted feeding TRF (16:8)	In TRF group, 8 h eating window followed by 16 h fasting window			TRF group also reduced weight more significantly than control group	
Kord Varkaneh et al. (2022)	44	Overweight, obese and NAFLD patients, age group: 18–50 years, BMI: 25–40 kg/m^2^, 17 females and 27 males	12 weeks	IF (5:2)	In IF (5:2), 25% calorie intake of the total energy requirements on two consecutive fasting days followed by normal food intake on remaining 5 days	Usual diet	−3.7 kg in IF (5:2) group, −1.02 kg in control group	IF (5:2) demonstrated significant body weight reductions in comparison with the control group	Not applied
Steger et al. (2023)	59	Obese, age group: 25–75 years, BMI: 30–60 kg/m^2^, 52 females and 20 males	14 weeks	Time restricted Eating + Energy Restriction (TRE + ER) At least 6 days a week	8 h eating window followed by 16 h fasting	CON+ ER (eating ≥ 12‐h self‐selected period) at least 6 days a week	−7.6 ± 1.0 kg in TRE + ER group −3.9 ± 0.9 kg in CON+ER group	Participants in TRE + ER group had greater improvements in weight than those in control group	Not applied
Schroder et al. (2021)	32	Obese women, age group not mentioned, BMI: ≥ 30 kg/m^2^, 32 females and 0 males	3 months	Time restricted fasting (16:8)	16 h fasting window followed by 8‐h ad libitum feeding without any kcal restriction	Habitual diet	−3.38 kg in TRE group +1.35 kg in Control group	TRE is effective in reducing body weight without changes in biomarkers related to metabolic syndrome	Not applied
Fadhadli et al. (2021)	36	Overweight and obese adults, age range: 20–50 years, BMI range: 25.0–43.0 kg/m^2^, 6 males and 6 females per group	16 weeks	Group A (TRF combined with Brisk Walk)	TRF combined with Brisk Walk (BW), in which TRF is 16‐h fasting period with an 8‐h ad libitum eating window for 5 days per week	Group C (control group maintaining their lifestyle)	TRF + BW: ΔBMI = −1.9 kg/m^2^, control ΔBMI = −0.59 kg/m^2^	There was no significant change in the body weight characteristics and fat parameters.	No significant difference has been observed between TRF + BW and TRF alone in terms of body weight and fat loss parameters
				Group B (TRF alone)	TRF is 16‐h fasting period with an 8‐h ad libitum eating window for 5 days per week		TRF alone: ΔBMI = −2.0 kg/m^2^, control: ΔBMI = −0.59 kg/m^2^		
Kotarsky et al. (2021)	21	Overweight or obese adults, age: 35–60 years, BMI: 25–34.9 kg/m^2^, 18 females and 3 males	8 weeks	TRE (16:8) + resistance training (RT)	The subjects fasted for 16 h and had eating window of 8 h with resistance training (three sets of 12 repetitions, with not more than 60 s rest between exercise and sets, at 1–7 p.m. on non‐consecutive days each week)	Normal eating+ resistance training (NE), with no exercise in fasted state	TRE (3.3% body mass loss), Control (0.2% reductions in body mass)	TRE group depicted significant improvement in body mass than control group	Not applied
Lowe et al. (2020)	105 (completed)	Overweight and obese participants, age group: 18–64 years, BMI: 27–43 kg/m^2^, 46 females and 70 males (enrolled)	12 weeks	TRE (16:8)	Fasting for 16 h (8–12 p.m.), followed by ad libitum meals for remaining 8 h (12–8 p.m.)	Conistent meal timing group (CMT: three structured meals a day)	−0.94 kg in TRE group, −0.68 kg in CMT group	No significant difference between the intervention and control group (−0.26 kg or −0.41%)	Not applied
Kunduraci and Ozbek (2020)	65	Adults with metabolic syndrome, age: 18–65 years, BMI: ≥ 25 kg/m^2^, 31 males and 34 females	12 weeks	Intermittent energy restriction (IER)	The group participants followed 16 h fasting with 8 h eating window (25% energy restriction of the daily energy needs in 8 h eating window)	Continuous energy restriction (CER: 25% energy deficit in daily requirements)	8% weight loss in IER group and 6% in CER group	Both IER and CER groups demonstrated similar weight loss (~7%)	Not applied
Cienfuegos et al. (2020)	49	Obese adults, mean age: 46 ± 2.3 years, BMI: 30–50 kg/m^2^, 44 females and 5 males	10 weeks	4‐h time restricted feeding (TRF)	2‐week weight stabilization period was followed by 8‐week intervention period during which the 4‐h TRF group was instructed to eat ad libitum from 3 to 7 p.m. daily and fast from 7 to 3 p.m. (20‐h fast).	Control was instructed to maintain their weight throughout the trial and not alter their eating habits and physical activity.	4‐h TRF group (∆ = **−**3.2% ± 0.4%), control (∆ = 0.1% ± 0.4%)	4‐h TRF group loses significantly more weight than control.	The study demonstrated that 4‐h and 6‐h TRF have significantly no difference between them in terms of weight loss.
				6‐h time restricted feeding (TRF)	2‐week weight stabilization period was followed by 8‐week intervention period during which the 6‐h TRF group was instructed to eat ad libitum from 1 to 7 p.m. daily and fast from 7 to 1 p.m. (18‐h fast).		6‐h TRF group (∆ = **−**3.2% ± 0.4%), control (∆ = 0.1% ± 0.4%)	6‐h TRF group shows significantly more weight loss than control group.	
Hutchison et al. (2019)	68	Overweight women, age: 35–70 years, BMI: 25–42 kg/m^2^, all females	10 weeks (2 weeks lead in phase and 8 weeks intervention phase)	Intermittent fasting IF (70)	The subjects consumed 32% of the energy requirements at breakfast (8 a.m.) and then fasted for 24 h until the next day (8 a.m.) on three non‐consecutive days a week while on fed days, they consumed 100% of the calorie requirements per week	No calorie restriction	No numerical value reported	IF (70) group found more weight reductions than control group	IF (70) demonstrated significant weight loss among all other intervention groups.
				Intermittent fasting IF (100)	In this group, the participants consumed 37% of the calorie requirements at breakfast (8 a.m.) and then fasted for 24 h until the next day, on three non‐consecutive days a week while on fed days, they consumed 145% of the energy requirements a week			IF (100) group depicted greater weight loss than control	
				Daily restriction DR (70)	The group participants followed 70% restriction of the calculated calorie needs			DR (70) group lost more weight than control	
Schübel et al. (2018)	136 (completed)	Overweight or obese participants, age: 25–40 years, BMI: 35–65 kg/m^2^, 50% females (enrolled)	50 weeks (12 weeks intervention, 12 weeks maintenance and 26 weeks follow‐up period)	Intermittent calorie restriction ICR (5:2)	The subjects followed 75% energy restriction on 2 days, followed by no calorie restriction on remaining 5 days	No energy restriction	ICR: −7.1% ± 0.7%, Control: −3.3% ± 0.6% (at the end of intervention period)	ICR group found greater weight reductions compared to the control	Both ICR and CCR demonstrated comparable results
				Continuous calorie restriction (CCR)	The subjects followed 20% energy deficit in daily energy needs		CCR: −5.2% ± 0.6% Control: −3.3% ± 0.6% (at the end of intervention period)	CCR group participants lost more weight in comparison with control group	
Trepanowski et al. (2017)	69	Obese adults, age group: 18–64 years, BMI: 25–39.9 kg/m^2^, 57 females and 12 males	1 year	Alternate day fasting (ADF)	In ADF group, 25% energy consumption on fasting day followed by 125% energy needs on feast days	Normal diet	ADF: −6.0% weight loss	ADF found better weight outcomes than control group	No significant difference in results between ADF and CCR group
				Continuous calorie restriction (CCR)	In CCR group, 75% of energy requirements each day		CCR: −5.3% weight loss	CCR participants also showed significant weight loss than control	
Bhutani et al. (2013)	64 (completed)	Overweight and obese, age group: 25–65 years, BMI: 30–39.9 kg/m^2^, 80 females and 3 males	12 weeks 4 weeks controlled feeding +8‐week self‐selected feeding period	Alternate day fasting (ADF)	In ADF group, 25% calories on fast days alternated with ad libitum meals on feast days	Ad libitum meals	−3 ± 1 kg weight from baseline weight in ADF group, 0 ± 0 kg in control group	ADF group participants lose greater weight than the control group	ADF+ exercise showed notable changes in weight when compared to ADF and exercise alone
				Exercise	In exercise group, ad libitum meals and moderate intensity exercise 3 times/week		−1 ± 0 kg change in weight from baseline weight in exercise group, 0 ± 0 kg in control group	Participants in exercise group demonstrated better results than control	
				ADF + Exercise	In ADF+ exercise group, one meal on fast day alternated with ad libitum meals on feast days and moderate intensity training program 3 times a week		−6 ± 4 kg change in weight from baseline in ADF+ exercise group, 0 ± 0 kg in control group	The ADF and exercise combined group outperformed the control group	
Varady et al. (2011)	49	Overweight or obese subjects, age group: 35–65 years, BMI: 25–39.9 kg/m^2^, 40 women and 9 men	12 weeks	Alternate day fasting (ADF)	In ADF group, 75% calories consumption for 24 h alternated with ad libitum feeding for 24 h	Usual diet	−5.2% ± 1.1% weight in ADF group‐0.2% ± 0.4% weight in control group	ADF found better weight outcomes than control	Both ADF and CCR showed similar degree of weight loss
				Continuous calorie restriction (CCR)	In CCR, 25% calories restriction each day		−5.0% ± 1.4% weight in CCR group −0.2% ± 0.4% weight in control group	The CCR group depicted more favorable results than control	

^a^ This column explicitly states the number of participants who completed the study.

^b^ This column states the number of males and females completing the study. In case if not provided by the authors, it reports this data for enrolled participants as per provided information in the article.

^c^ The units for outcome measurement show heterogeneity. Weight reduction is presented in kg or % as documented in the original studies. In studies where direct weight change was not reported, alternative anthropometric outcomes (e.g., body mass or BMI) are presented, as reported in the original studies.

Information on the provision of calorie‐restricted meals and the inclusion of counseling or behavioral support sessions was also documented. Follow‐up durations, participant adherence, sources of funding, and the outcomes of bias assessments were further extracted and reported for each included study. Body weight was recorded as the primary outcome across the studies for both intervention and control groups, measured either in kilograms or as a percentage change from baseline. For the studies having no measurement of body weight, BMI and additional body composition parameters, including fat mass, lean mass, visceral fat mass, percentage body fat, and waist circumference, were evaluated and reported.

### 2.6 Synthesis

All studies included in the synthesis had fulfilled the pre‐established eligibility criteria during the screening process. Key study characteristics, including author names, publication year, duration, sample size, study design, intervention and control details, and reported outcomes, were compiled in a tabular format by a single researcher. No graphical representations (e.g., charts or plots) were generated for this tabulated data. The studies were analyzed narratively and the subgroup analysis couldn't be undertaken, due to variations in different IF interventions types (ADF, TRE, TRE + RT, ADF+ exercise, CCR), comparators (usual diet, ad libitum meals, routine lifestyle, control+ energy restriction), intervention arms and limited studies for each subgroup. A summary of weight loss among different forms of intermittent fasting is presented in Table 3. There were no conversions or transformations, and no imputations in all data extracted and all results presented narratively; no sensitivity analysis was conducted to evaluate the robustness or variability of the synthesized results.

**TABLE 3:** Comprehensive summary of weight loss outcomes of interventions.

IF category	Study (author, year)	Duration range	Protocol specifications	Reported weight or BMI change (kg or %)
*Alternate day fasting (ADF)*				
ADF alone	Ezpeleta et al. (2023)	3 months	600 kcal consumption as a dinner on fast days	−5.6%
	Chair et al. (2022)	3 weeks	600 kcal consumption on fasts day	Significant weight loss reported (numerical value not provided)
	Trepanowski et al. (2017)	1 year	25% energy consumption on fast day and 125% on feast days	−6.8%
	Bhutani et al. (2013)	12 weeks	25% calorie consumption on fast days	−3 ± 1 kg
	Varady et al. (2011)	12 weeks	75% calorie consumption on fast days	−5.2% ± 1.1%
	Hutchison et al. (2019)	8 weeks	IF 70 (24 h fast followed by 12 h eating window on three non‐consecutive days, 32% energy intake as breakfast on fast day)	Significant weight loss reported (Numerical value not provided)
	Hutchison et al. (2019)	8 weeks	IF 100 (24 h fast followed by 12 h eating window on three non‐consecutive days, 37% energy intake as breakfast on fast day, overall, 145% energy provided)	— (Numerical value not provided)
ADF with exercise	Ezpeleta et al. (2023)	3 months	600 kcal consumption as dinner on fast days and participation in moderate‐intensity aerobic exercise 5× per week for 3 months	−4.58%
	Bhutani et al. (2013)	12 weeks	One meal on fast day & moderate intensity training program 3 times a week	−6 ± 4 kg
ADMF (alternate day modified fasting)	Hooshiar et al. (2023)	8 weeks	25% energy deficit on fasting days & 100% energy consumption on feast days	−5.23 kg
	Martínez‐Montoro et al. (2025)	3 months	24 h fasting, 4 days per week, with 600 kcal energy deficit	−11.8 kg
Time restricted eating/feeding (TRF/TRE) 16:8				
TRF (16:8) alone	Oldenburg et al. (2025)	12 weeks	Fasting for 16 h, with 8 h eating window in a day without calories restriction	−3.0 kg
	Rizvi et al. (2024)	12 weeks		ΔBMI% = 10.04 ± 4.15
	Domaszewski et al. (2023)	6 weeks		−1.8 kg in males and −1.3 kg in females
	Lin et al. (2023)	12 months (6 months weight loss and 6 months weight maintenance phase)	16 h of fasting with 8 h eating window in weight loss phase, 14 h fast followed by 10 h eating in weight maintenance phase	−3.49 kg
	Chair et al. (2022)	3 weeks	16‐h fast with 8‐h eating window (without calories restriction)	— (Numerical value not provided).
	Schroder et al. (2021)	3 months		−3.38 kg
	Lowe et al. (2020)	12 weeks		−0.94 kg
	Fadhadli et al. (2021)	16 weeks	16‐h fast with 8 h ad libitum eating, 5 days a week	ΔBMI = −2.0 kg/m^2^
	Steger et al. (2023)	14 weeks	16 h fast and early 8 h eating window at least 6 days/week, with calorie restriction of 500 kcal/day	7.6 ± 1.0 kg (7.0% ± 0.9%)
	Kunduraci and Ozbek (2020)	12 weeks	16 h of fasting with 25% energy restriction of daily energy requirements in 8 h eating window	−8%
	Martínez‐Montoro et al. (2025)	3 months	16 h fast with early 8 h eating window, consuming three meals with 600 kcal deficit	−9.4 kg (−10.4 to −8.5 kg)
	Martínez‐Montoro et al. (2025)	3 months	16 h fast with late 8 h eating window, consuming three meals with 600 kcal deficit	−10.6 kg (−11.7 to −9.4 kg)
TRF (16:8) with exercise	Fadhadli et al. (2021)	16 weeks	16 h fast with 8 h ad libitum eating, 5 days a week with 1 h of continuous brisk walking for 5 days a week.	−1.9 kg/m^2^
	Kotarsky et al. (2021)	8 weeks	16 h fast with 8 h eating window, in combination with resistance training on non‐consecutive days	−3 kg
TRE 16:8 combined with diet	Shafiee et al. (2025)	12 weeks	16 h and 8 h eating in a day, with LOV diet followed in 8 h eating window	−8.07 ± 4.31 kg (−8.9%)
Intermittent fasting 5:2				
IF (5:2) alone	Witjaksono et al. (2022)	8 weeks	Fasting for 2 days followed by ad libitum meals on the remaining days of week	−0.8 kg
	Kord Varkaneh et al. (2022)	12 weeks	25% of the caloric intake on two fast days, and no restriction on rest of the days of week	−3.7 kg
	Schübel et al. (2018)	50 weeks	75% energy restriction on 2 days, with ad. Libitum meals on remaining 5 days of a week	−7.1% ± 0.7%
Intermittent fasting 14:10				
TRE (14:10)	Quist et al. (2024)	6 months	Self‐selected 10 h eating period followed by 14 h fasting	−1.1 kg
	Tsitsou et al. 2025	12 weeks	14 h fast, early 10 h eating window, with 500 kcal deficit	−7.2 ± 3.3 kg
	Tsitsou et al. 2025	12 weeks	14 h fast, late 10 h eating window, with 500 kcal deficit	−7.1 ± 3.7 kg
TRE 14:10 with supplementation	Talebi et al. (2024)	8 weeks	14 h fast, early 10 h eating window, with probiotic supplementation	−2.2 ± 1.6 kg
Other types of intermittent fating				
TRE (20:4)	Cienfuegos et al. (2020)	10 weeks	20 h fasting followed by ad libitum eating for 4 h	−3.2% ± 0.4%
TRE (18:6)	Cienfuegos et al. (2020)	10 weeks	18 h fasting followed by ad libitum eating for 6 h	−3.2% ± 0.4%

### 2.7 Bias Assessment

The quality appraisal of the included studies was done by two different researchers, and their results were cross‐verified through a structured discussion process and consensus. The quality assessment was also conducted repeatedly to enhance transparency and increase the reliability and validity of the results. Of the 26 included randomized controlled trials, 24 used the revised Cochrane Risk of Bias 2.0 tool (RoB2) to evaluate methodological quality (Sterne et al. 2019). The tool assesses five areas of potential bias, including evaluation of the randomization process, variations due to planned interventions, missing outcome data, measurement of the outcome, and the final choice of the reported outcome. The general risk of bias for each article was determined to be low risk, some concerns, or high risk, depending on the domain‐level judgment using the Cochrane Risk of Bias 2.0 tool (RoB2).

In addition, two non‐randomized included studies were assessed using non randomized study domain of the Mixed Methods Appraisal Tool (MMAT) (Hong et al. 2018), well‐designed for quality assessment across multiple study designs (Schroder et al. 2021; Fadhadli et al. 2021). The methodological questions of the Mixed Methods Appraisal Tool (MMAT) has five questions per domain of the study. Through MMAT, the methodological investigation in the non‐randomized study covered a well‐representative target population, appropriateness of measurement devices, completeness of outcome measures, control of confounding factors, and fidelity of intervention delivery. The risk‐of‐bias assessment was done after the inclusion and was not regarded as a criterion of the study inclusion. All the studies having moderate or high risk were interpreted with caution, while drawing the overall findings.

## 3 Results

### 3.1 Study Selection

**FIGURE 1:**
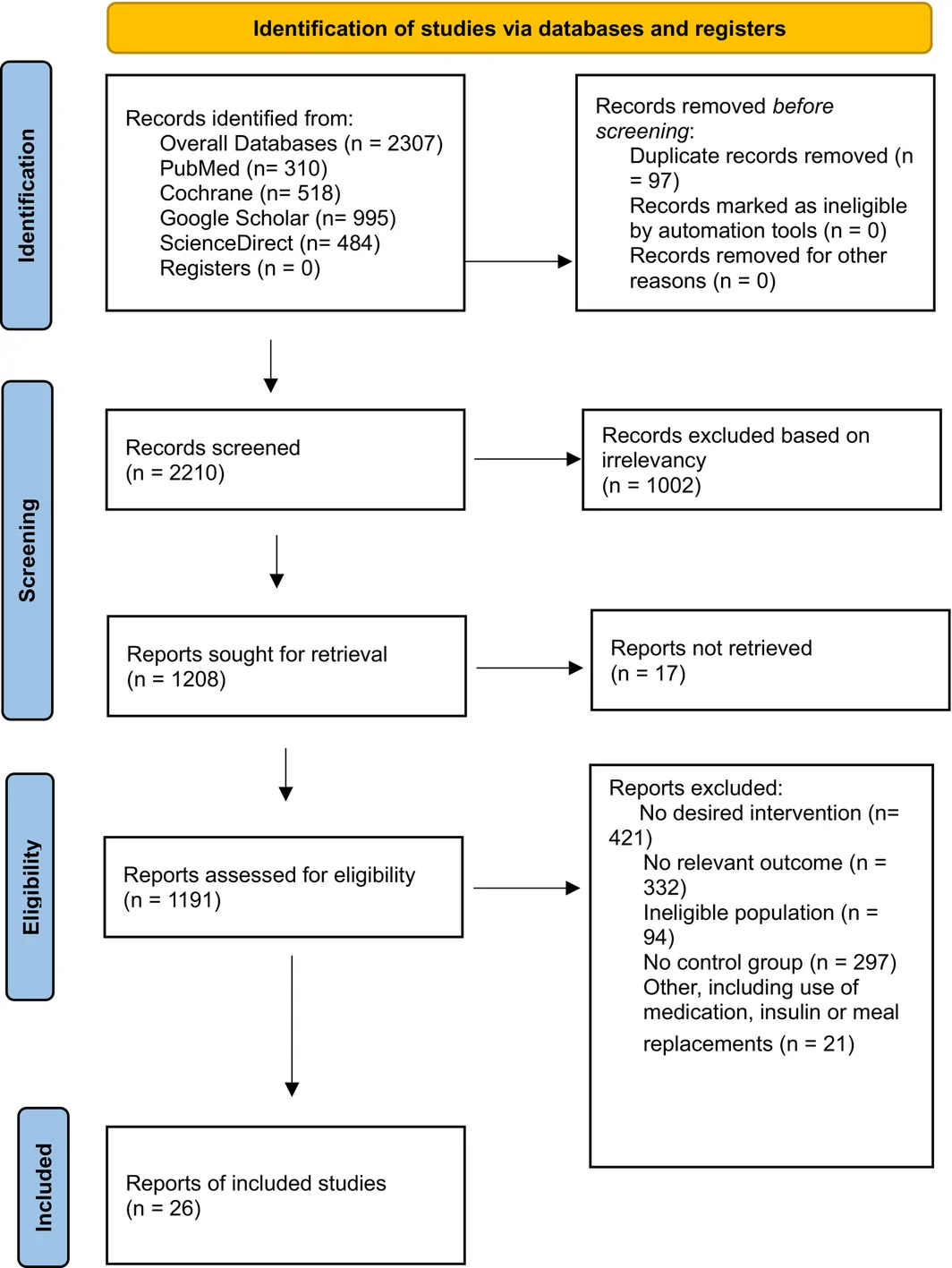
Search and study selection for systematic reviews (PRISMA) flow chart.

Two thousand three hundred seven studies were identified through the four databases. After removing duplicates, 2210 articles remained. The titles and abstracts were screened by three independent reviewers, resulting in 1208 studies that were selected for full‐text review. A large proportion of irrelevant records were retrieved from Google Scholar due to the use of broad search fields (searching “anywhere in the article”), which increased sensitivity but also resulted in the inclusion of studies not directly related to the topic. In addition, studies were excluded based on predefined eligibility criteria, including observational studies, pilot studies, review articles, in vivo studies and protocols. Ultimately, 26 articles met all inclusion criteria and were selected for this systematic review. Discrepancies were resolved through discussion among reviewers. This selection process is illustrated in the PRISMA flow diagram (Figure 1).

### 3.2 Characteristics of Study

Among the 26 studies that were utilized, 24 were randomized controlled trials (Oldenburg et al. 2025; Shafiee et al. 2025; Talebi et al. 2024; Martínez‐Montoro et al. 2025; Tsitsou et al. 2025; Rizvi et al. 2024; Quist et al. 2024; Ezpeleta et al. 2023; Hooshiar et al. 2023; Lin et al. 2023; Steger et al. 2023; Domaszewski et al. 2023; Chair et al. 2022; Kord Varkaneh et al. 2022; Witjaksono et al. 2022; Kotarsky et al. 2021; Lowe et al. 2020; Kunduraci and Ozbek 2020; Cienfuegos et al. 2020; Schübel et al. 2018; Hutchison et al. 2019; Trepanowski et al. 2017; Bhutani et al. 2013; Varady et al. 2011). Two studies were non‐randomized controlled trials (Schroder et al. 2021; Fadhadli et al. 2021). All the studies had a control group, along which the respective intermittent fasting interventions were compared to comparators that included standard diet, exercise, calorie restriction, or combinations of these. Study durations ranged from 3 to 50 weeks, and publication years spanned from 2011 to 2025. Interventions included multiple IF types—ADF, Warrior Diet, 16:8, 20:4, 5:2, and 18:6 regimens. Nineteen studies disclosed funding sources (Oldenburg et al. 2025; Talebi et al. 2024; Martínez‐Montoro et al. 2025; Quist et al. 2024; Ezpeleta et al. 2023; Hooshiar et al. 2023; Steger et al. 2023; Lin et al. 2023; Witjaksono et al. 2022; Kord Varkaneh et al. 2022; Kotarsky et al. 2021; Schroder et al. 2021; Lowe et al. 2020; Cienfuegos et al. 2020; Schübel et al. 2018; Trepanowski et al. 2017; Hutchison et al. 2019; Bhutani et al. 2013; Varady et al. 2011).

### 3.3 Risk of Bias Assessment

A comprehensive bias assessment was conducted among all the included studies to evaluate their methodological quality. The studies included were primarily those with a single control group and two to three intervention arms. The bias assessment involved comparisons of each intervention group alone (excluding the control group) with the control group. RoB2 indicated that among the 24 included RCT studies, one (Steger et al. 2023) was assessed as having a high risk of bias (bias due to deviations from intended intervention and missing outcome data), and 16 were determined to have low risk. While the remaining 7 RCTs showed some concerns (moderate risk), resulting in decreased confidence in the obtained results. Among these 24 randomized controlled trials, the bias arose from randomization process, causing selection bias in three studies (Domaszewski et al. 2023; Kunduraci and Ozbek 2020; Trepanowski et al. 2017). In two studies, the deviations occurred from the intended intervention, leading towards performance bias (Shafiee et al. 2025; Talebi et al. 2024). The bias occurred in the selection of reported results in a study by Bhutani et al. (2013). Lastly, one study demonstrated the bias due to deviations in randomization process and selection of reported results (Varady et al. 2011). The findings of the studies having some concerns or high risk were interpreted with caution while drawing conclusions. A summary of these findings, presented in a visual form as a traffic light plot, is provided in Table 4. The detailed RoB2 assessment form for these 24 studies is available in Supporting Information: Appendix A.

**TABLE 4:**
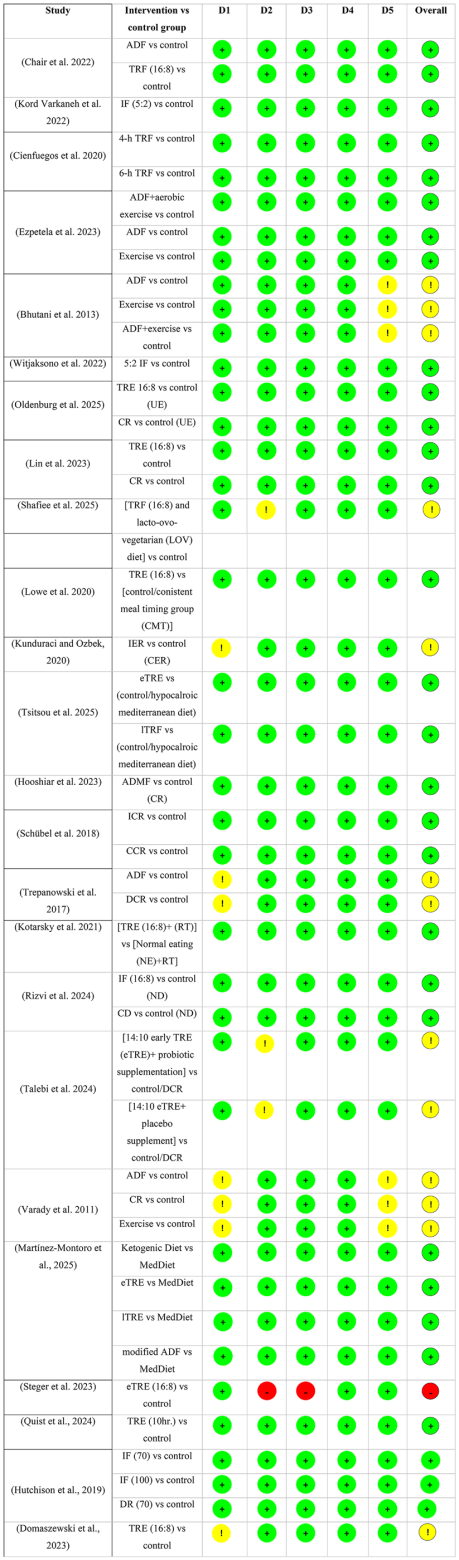
Risk of bias assessment of studies using RoB‐2 (*n* = 24).

*Note:* Here, D stands for Domain. D1: Randomization Process, D2: Deviations from the intended interventions, D3: Missing outcome data, D4: Measurement of the outcome, D5: Selection of the reported result, overall: Overall bias of the study. Judgment = Green (+): Low risk, Yellow (!): Some concerns and Red (−): High risk. Each row indicates the bias assessment of a specific intervention group vs. control from a study.

While the two non‐randomized included studies were appraised under non‐randomized controlled studies criteria of the MMAT tool. The results demonstrated that one study satisfied 3 out of 5 criteria while the other study fulfilled 2/5 quality assessment criteria, as evidenced in Table 5. A complete MMAT assessment form is provided in Supporting Information: Appendix B.

**TABLE 5:** MMAT bias assessment checklist.

Study	Quality assessment criterion				
	S1	S2	S3	S4	S5
Schroder et al. (2021)	Yes	Yes	Yes	No	No
Fadhadli et al. (2021)	No	Yes	No	Yes	Can't tell

*Note:* S1: Are the participants representative of the target population? S2: Are measurements appropriate regarding both the outcome and intervention (or exposure)? S3: Are there complete outcome data? S4: Are the confounders accounted for in the design and analysis? S5: During the study period, is the intervention administered (or exposure occurred) as intended?

### 3.4 Participants

Across all included studies, a total of 1847 participants were involved who completed all aspects of trials. Sample sizes ranged from 21 (Kotarsky et al. 2021) to 140 (Martínez‐Montoro et al. 2025), with 21 studies enrolling fewer than 100 participants. Female participants constituted the majority, that is, 1326 (70.87%), while males accounted for 545 (29.13%), including those who dropped out, for studies which did not state the gender distribution for the completers.

Out of 26 included studies, only one study involved intermittent Calorie Restriction (ICR), that is, 5:2 type (Schübel et al. 2018). Six studies contained a group with calorie restriction compared with the intervention or control group and named as CCR (Continuous Calorie Restriction), DCR (Daily Calorie Restriction), DR (Dietary Restriction) or CR (Calorie Restriction). Four studies contained energy‐restricted control diets (Tsitsou et al. 2025; Martínez‐Montoro et al. 2025; Talebi et al. 2024; Hooshiar et al. 2023; Steger et al. 2023), three studies contained exercise‐only regimens along with other IF types (Bhutani et al. 2013; Ezpeleta et al. 2023; Varady et al. 2011), 13 studies followed TRE with calorie restriction, and three studies combined TRE with exercise (Ezpeleta et al. 2023; Kotarsky et al. 2021; Bhutani et al. 2013). The mean age ranged from 28.3 ± 6.3 years (Fadhadli et al. 2021) to 69 ± 3.46 years (Domaszewski et al. 2023), while the range of mean body weight was 74.30 kg (Chair et al. 2022) to 107.5 ± 19.5 kg (Martínez‐Montoro et al. 2025), except those studies which didn't directly state the mean weight at baseline. Only one study included participants with pre‐diabetes (Chair et al. 2022), two studies included participants with Non Alcoholic Fatty Liver Disease (NAFLD) (Ezpeleta et al. 2023; Kord Varkaneh et al. 2022), two studies involved patients with Metabolic Associated Fatty Liver Disease (MAFLD) (Shafiee et al. 2025; Tsitsou et al. 2025) while only one study included T2DM and HTN patient along with healthy participants (Schroder et al. 2021). Twenty‐one studies did not report any statistically significant baseline group differences. Chair et al. (2022) found statistically significant differences in gender (*p* = 0.013), marital status (*p* = 0.025), HDL‐C (*p* = 0.027), total cholesterol (p = 0.006), and physical activity level (*p* = 0.003), which were then adjusted while conducting GEE analysis. Domaszewski et al. (2023) reported that relative fat mass and BMI were higher in the females of experimental group at baseline, for which pairwise Tukey follow‐up tests were performed to pinpoint the differences and adjust them. Similarly, Talebi et al. (2024) reported statistical difference in the age of onset of menstruation among the three groups (*p* = 0.036), which were adjusted through general linear models (GLM). Baseline imbalances between groups for blood pressure and the Baecke total physical activity score (*p* ≤ 0.04) were also adjusted in the study of Steger et al. (2023). Dropout rates varied from 0.99% (Chair et al. 2022) to 34.4% (Steger et al. 2023) mainly due to scheduling conflicts, personal reasons, or participant inaccessibility.

### 3.5 Dietary Protocol

Considerable heterogeneity existed in the dietary protocols across 26 studies. Seven studies utilized alternate‐day fasting (ADF) (Martínez‐Montoro et al. 2025; Ezpeleta et al. 2023; Hooshiar et al. 2023; Chair et al. 2022; Trepanowski et al. 2017; Bhutani et al. 2013; Varady et al. 2011), with one study employing a 25% caloric intake on fasting days and specifying 125% on feast days (Trepanowski et al. 2017). Bhutani et al. (2013) reported a 4‐week controlled feeding period and an 8‐week self‐selected feeding period, consuming 25% of their baseline energy needs on the “fast day.” A study by Hooshiar et al. (2023) reported that the group following Alternate Day Modified Fasting (ADMF) intervention consumed 25% of energy needs on fast days. Martínez‐Montoro et al. (2025) adjusted the calories such that participants consumed 25%–30% of total energy requirements or ~400–800 kcal/day, 3 days per week. Moreover, Chair et al. (2022) and Ezpeleta et al. (2023) reported that participants consumed 600 kcal on fasting days. While Hutchison et al. (2019) provided IF70 participants with ~100% on fed days and IF100 with ~145% of required energy.

Cienfuegos et al. (2020) adopted time‐restricted eating (TRE) with 4‐h or 6‐h eating windows, allowing ad‐libitum intake during feeding and only water or non‐caloric beverages during fasting. Kord Varkaneh et al. (2022) implemented a 5:2 fasting regimen, where participants reduced their energy intake to 25% on two consecutive fasting days per week while Witjaksono et al. (2022) subjected the participants to 5:2 intermittent fasting regimen with no calorie restriction and fast for ±14 h. Ten studies employed the 16:8 protocol; two allowed regular meals during eating windows (Lin et al. 2023; Schroder et al. 2021), while one included a daily caloric restriction of 500 kcal (Steger et al. 2023). Among other studies that employed 16:8 type, Kunduraci and Ozbek (2020) reported 25% energy restricted diet in 16:8 model of Intermittent Energy Restriction (IER) intervention group. Martínez‐Montoro et al. (2025) implemented early TRE with 8‐h eating window between 8 a.m. and 4 p.m. while late TRE between 2 p.m. and 10 p.m. Shafiee et al. (2025) followed 200–500 kcal less than the energy needed for maintenance per day in the intervention group. However, three studies reported IF 14:10 intervention with 10 h of eating window (Tsitsou et al. 2025; Quist et al. 2024; Talebi et al. 2024).

Calorie restriction alone groups showed variability across different studies: Oldenburg et al. (2025) implemented 15% energy restriction; one applied 20% energy restriction daily (Schübel et al. 2018); four applied 25% restriction (Lin et al. 2023; Kunduraci and Ozbek 2020; Trepanowski et al. 2017; Varady et al. 2011). Hooshiar et al. (2023) study involved 63% daily calorie consumption; Hutchison et al. (2019) applied 70% daily energy intake. Lin et al. (2023) used cognitive‐behavioral techniques to support weight control. Thirteen studies described detailed macronutrient compositions with slight variations (Tsitsou et al. 2025; Shafiee et al. 2025; Martínez‐Montoro et al. 2025; Talebi et al. 2024; Hooshiar et al. 2023; Ezpeleta et al. 2023; Lin et al. 2023; Kord Varkaneh et al. 2022; Witjaksono et al. 2022; Hutchison et al. 2019; Trepanowski et al. 2017; Bhutani et al. 2013; Varady et al. 2011).

### 3.6 Food Selection

Only one study supplied calorie‐restricted meals to ADF participants for the entire study duration (Varady et al. 2011). Trepanowski et al. (2017) provided meals for the first 3 months of a 12‐month study, in accordance with American Heart Association guidelines (AHA). Bhutani et al. (2013) delivered meals during a 4‐week controlled feeding phase to ADF and ADF‐plus‐exercise groups. Moreover, Ezpeleta et al. (2023) offered meals to intervention group for fasting days during the 1st month of the study. In a study by Martínez‐Montoro et al. (2025), two groups, that is, modified alternate day fasting and ketogenic diet group were supported by commercial meal replacement diets. Ten studies provided dietary counseling (Martínez‐Montoro et al. 2025; Ezpeleta et al. 2023; Lin et al. 2023; Steger et al. 2023; Chair et al. 2022; Kord Varkaneh et al. 2022; Witjaksono et al. 2022; Schübel et al. 2018; Bhutani et al. 2013; Varady et al. 2011). Personalized diet plans were given to intervention groups in five studies (Martínez‐Montoro et al. 2025; Shafiee et al. 2025; Domaszewski et al. 2023; Kord Varkaneh et al. 2022; Schübel et al. 2018). However, in one study, the IF group was compared with the customized diet plan group, resulting in comparable weight loss (Rizvi et al. 2024). Three studies offered personalized diet menus to the intervention groups (Martínez‐Montoro et al. 2025; Chair et al. 2022; Kunduraci and Ozbek 2020).

### 3.7 Exercise

Only three studies evaluated the effect of exercise either independently or in combination with intermittent fasting. Ezpeleta et al. (2023) and Bhutani et al. (2013) assessed the impact of combining Alternate Day fasting (ADF) with exercise, while Varady et al. (2011) compared ADF, CR, exercise, and control groups. Exercise regimens involved moderate‐intensity training three times weekly, although the intensity and duration varied. Ezpeleta et al. (2023) and Bhutani et al. (2013) observed significant weight loss in the ADF‐plus‐exercise group. Varady et al. (2011) found no significant differences among groups.

### 3.8 Weight Loss Outcomes

All studies demonstrated significant weight reductions over durations ranging from 3 weeks (Chair et al. 2022) to 50 weeks (Schübel et al. 2018).

Studies combining IF with exercise generally reported significant weight loss in comparison with the control group. For instance, Bhutani et al. (2013) found greater weight loss in the combination of ADF and moderate intensity exercise program (−6 ± 4) as compared to ADF alone (−3 ± 0 kg) or exercise alone (−1 ± 0 kg). Ezpeleta et al. (2023) also found notable weight loss in the ADF + exercise group, that is (−4.58%), as compared to exercise alone, that is (−1.30%), but no significant difference in the weight loss results of ADF + exercise and ADF alone group (−5.06%) was found. Hutchison et al. (2019) reported that weight loss was significantly higher in the IF70 group compared with the DR70 and IF100 group (*p* ≤ 0.05). Chair et al. (2022) reported better outcomes in ADF group compared to the 16:8 TRE. Trepanowski et al. (2017) study showed sustained weight loss with ADF and CR at both 6‐ and 12‐month intervals. Furthermore, IF (5:2) documented greater improvements in body weight (−3.7 kg) in comparison with the control group (−1.02 kg) (Kord Varkaneh et al. 2022). The study by Witjaksono et al. (2022) indicated although −0.8 kg weight loss occurred in IF (5:2) group and −0.3 kg in control group, but these results might not be significant from clinical perspective. The study proved that intermittent fasting 5:2 results in weight loss but with no large significant effects on other body composition parameters. Cienfuegos et al. (2020) reported no significant difference between the weight loss outcomes of 4 and 6 h TRF. Tsitsou et al. (2025) found no statistically significant variations in the weight outcomes of late and early TRF (14:10) after 12 weeks. The study by Talebi et al. (2024) reflected no significant differences in weight loss between eTRE (14:10) + probiotic supplementation and DCR with placebo supplementation group (−2.2 ± 1.6 kg in eTRE+ probiotic group, −2.5 ± 1.7 kg in DCR group).

Shafiee et al. (2025) found that 16:8 TRF with LOV‐D (lacto‐ovo‐ vegetarian) diet involving calories restriction resulted in 8.9% decrease in weight from the baseline (−8.07 ± 4.31 kg, *p* < 0.001) compared to a 4.2% decrease in the control group (−3.75 ± 3.97 kg, *p* = 0.002). Steger et al. (2023) observed decrease of 7.6 ± 1.0 kg in eTRE (16:8) + ER group while 3.9 ± 0.9 kg in control with energy restriction group, highlighting the positive effect of early TRE compared to a usual diet. Fadhadli et al. (2021) found that there were no significant differences between TRF (16:8) + BW and TRF (16:8) alone in terms of body weight and fat loss parameters. Martínez‐Montoro et al. (2025) observed a descending order of weight loss in the groups, that is, mADF > late TRE > early TRE > KD (Ketogenic diet) > control (MedDiet). An improvement in weight in the TRE 16:8 (−0.94 kg; 95% CI; *p* = 0.01) was observed, but there was no significant change in the Consistent Meal Timing group (−0.68 kg; *p* = 0.07) as well as no difference in these two groups (−0.26 kg; 95% CI; *p* = 0.63) (Lowe et al. 2020). In Schroder et al. (2021), 45% of women in the 16:8 group failed to lose more than 4% of their initial weight.

### 3.9 Body Composition Outcomes

Twenty‐two studies assessed different body composition markers beyond weight loss, including BMI, fat mass, lean mass, waist circumference, waist to hip ratio (WHR) and percentage body fat. Dual‐energy X‐ray absorptiometry was employed in seven studies (Oldenburg et al. 2025; Quist et al. 2024; Ezpeleta et al. 2023; Lin et al. 2023, Kotarsky et al. 2021; Lowe et al. 2020; Trepanowski et al. 2017). A bioelectrical impedance analyzer was utilized in two studies (Tsitsou et al. 2025; Bhutani et al. 2013). Four studies measured body composition using a TANITA monitor (Martínez‐Montoro et al. 2025; Kord Varkaneh et al. 2022; Witjaksono et al. 2022; Kunduraci and Ozbek 2020), and one study employed NHANES‐based anthropometric equations (Schroder et al. 2021). Furthermore, Domaszewski et al. (2023) and Fadhadli et al. (2021) used SECA mBCA 515 and OMRON fat analyzer to estimate the body composition measurements respectively. Hooshiar et al. (2023) and Chair et al. (2022) reported greater BMI reduction in ADF compared to the control group. Similarly, co‐intervention groups such as TRE combined with probiotic supplementation and ADF combined with exercise revealed improved body composition markers than the IF intervention groups alone (Ezpeleta et al. 2023; Talebi et al. 2024). Six included studies have found a significant reduction in fat mass in different IF groups (5:2, 14:10, 16:8 and IF with calorie restriction) compared to the control group (Quist et al. 2024; Lin et al. 2023; Domaszewski et al. 2023; Kord Varkaneh et al. 2022; Kotarsky et al. 2021; Kunduraci and Ozbek 2020; Hooshiar et al. 2018). Different authors have documented notable decreases in waist circumference in the TRF group (Martínez‐Montoro et al. 2025; Oldenburg et al. 2025; Tsitsou et al. 2025; Witjaksono et al. 2022; Schroder et al. 2021; Lowe et al. 2020). No significant differences were observed in fat‐free mass, lean mass, or visceral fat mass between intervention groups (Shafiee et al. 2025; Rizvi et al. 2024; Steger et al. 2023; Fadhadli et al. 2021; Trepanowski et al. 2017).

### 3.10 Compliance

One study lacked adherence data (Varady et al. 2011). Fourteen studies used food logs, digital records, checklists, questionnaires, dietary recalls, online forms, electronic survey, or adherence checklists. Chair et al. (2022), Schroder et al. (2021), and Lin et al. (2023) reported 87% adherence in the TRE group and 61% in the CR group, as measured by the ASA‐24. Trepanowski et al. (2017) noted that ADF participants consumed less than prescribed at Months 3, 6, 9, and 12. Kord Varkaneh et al. (2022) conducted monthly interviews, weekly phone calls, and 24 h dietary recalls for participants to adhere to the intervention. Kotarsky et al. (2021) reported the use of Food Processor software (ESHA) used by research assistants and line‐by‐line verified by a registered dietitian for analysis food records obtained and reported high level of compliance. Trepanowski et al. (2017) reported the use of Nutritionist Pro software (Axxya Systems LLC) to measure dietary intake and adherence. Oldenburg et al. (2025) used mCC app to record oral intake. Schübel et al. (2018) reported that direct measure of dietary intake could not be performed; however, the data on weight loss outcomes and changes observed in dietary intake indicated good compliance. In contrast, Lowe et al. (2020) indicated that 16:8 group showed high self‐reported adherence. In the study of Lin et al. (2023), participants in the TRE group were adherent on average 6.1 days per week, while for the CR group, 61% of participants reported being adherent. Hutchison et al. (2019) reported an important finding that IF 100 was perceived more difficult to be followed or adhered to than IF 70. Similarly, an important point was highlighted by Domaszewski et al. (2023) that older adults showed high adherence from 84% to 98% as they followed TRE 16:8.

### 3.11 Follow‐Up

Four studies incorporated follow‐up periods and two studies involved weight maintenance phases. A short‐term study with a three‐month follow‐up period compared the effects of alternate day fasting (ADF) with time restricted feeding (TRF 16:8). At follow‐up, statistically significant decreases in weight and BMI were observed in the ADF group. By the conclusion of the intervention, participants who completed the ADF‐based group lost 10.16 kg with a small positive change to 10.34 kg in the follow‐up (*p* < 0.001). BMI also decreased from −2.10 to −2.15 kg/m^2^ (*p* < 0.001). However, reductions in waist circumference were not statistically significant, ranging from 2.60 cm at the intervention's end to 2.65 cm at follow‐up (*p* = 0.199) (Chair et al. 2022). In addition, it was found that from the end of intervention period to follow‐up period, body weight between TRE participants and control was comparable (−0.8 kg difference) (Quist et al. 2024). Likewise, Tsitosou et al. (2024) and Schübel et al. (2018) observed no significant differences among the intervention groups, that is, eTRE and lTRE (14:10) with Mediterranean diet; ICR and CCR groups respectively at the end of follow‐up period. Moreover, Trepanowski et al. (2017) found that there were no significant differences in weight loss between intervention groups (ADF: −6% and DCR: −5.3%). The weight regains from Month 6 (end of intervention phase) to Month 12 (end of weight maintenance phase) was also non‐significant (−0.8%). Likewise, Lin et al. (2023) divided the study duration into two phases: 6 months weight loss phase and 6 months weight maintenance phase. At 12th month, both the intervention groups, that is, TRE and CR reported weight loss (TRE: −4.87% and CR: −5.30%) as compared to the baseline but no significant differences were observed between both intervention groups (0.43%).

## 4 Discussion

### 4.1 Weight Loss

The review of outcomes from 26 studies indicates that intermittent fasting can be a feasible and safe strategy for weight loss in overweight and obese adults. Numerous studies have exhibited that IF group participants lose more weight relative to the baseline. However, when comparing these different approaches of intermittent fasting (ADF, TRE 16:8, TRF 14:10, 4 h or 6 h TRE and ICR 5:2) with continuous calorie restriction (CCR), the trials demonstrated overlapping results (Oldenburg et al. 2025; Lin et al. 2023; Schübel et al. 2018; Trepanowski et al. 2017; Varady et al. 2011). Furthermore, a 12‐month study was divided into two phases: a 6‐month weight loss phase and a 6‐month maintenance phase. By Month 12, significant reductions in body weight were observed in both the TRE (16:8) group (4.87%, 95% CI: −7.61, −2.13) and the calorie restriction group (5.30%, 95% CI: −9.06, −1.54). However, the direct comparison of these two groups reported no statistically significant difference in weight loss (Lin et al. 2023). Schübel et al. (2018) reported a slight weight gain in the ICR (5:2) diet group compared to the CCR group initially. The study concluded that no significant difference was found in the weight loss in the two groups and both of these strategies can be easily followed and beneficial for obese and overweight population. The same study reported that for weight maintenance, 6:1 diet was feasible and sufficient instead of following 5:2 ICR diet for this purpose. Trepanowski et al. (2017) pointed out that ADF has long been assumed to be better than daily calorie restriction for weight loss. Instead, the study found that participants in the ADF group converted their diet into de facto calorie restriction as the time passed. A decline in adherence due to dissatisfaction with the diet was also observed among the study participants. Talebi et al. (2024) also found during the 8‐week trial that there was no significant variation in the weight loss results of early TRE with or without probiotics and daily calorie restriction. Similarly, the study by Oldenburg et al. (2025) reported no significant difference in the weight loss outcomes of 16:8 TRE and 15% daily CR group during 12 weeks. It further stated that self‐selected 8 h fasting is not sufficient to improve metabolic flexibility while long term short eating window is more beneficial to lose weight than practicing long hours fasting which can further decrease adherence.

In contrast, Kunduraci and Ozbek (2020) noted a significant difference in the weight loss means of IER (16:8) and CER both following energy restriction of 25%. Similarly, IF 70 group had significantly greater weight loss than DR 70 group as reported by Hutchison et al. (2019). The differences in different parameters among the studies such intervention period, energy restriction levels, sample sizes, adherence level, drop outs, follow ups, and differences in monitoring levels may influence the outcomes observed in these studies. Hooshiar et al. (2023) also reported significant difference in weight loss in the ADMF group (involving 75% calories restriction on fast days) as compared to the CR group (63% energy consumption day). In this case, ADF was combined with calorie restriction and produced better results than CR.

These results align with the previous studies which reported comparable effectiveness for a longer period (12 months) between these two approaches. Overall, the evidence indicates that intermittent fasting may be as effective as continuous calorie restriction in achieving long‐term weight management. In addition, intermittent fasting can serve as an alternative dietary intervention rather than superior weight loss strategy (Headland et al. 2019; Sundfør et al. 2018). Furthermore, IER and CER, resulting in at least a 5% weight loss, did not affect the satiety quotient; they modestly decreased hunger and the desire to eat (Beaulieu et al. 2020). As a consequence, they help to further reduce calorie consumption, and this effect ultimately potentiates the weight loss. Sundfør et al. (2018) also stated that adherence in the intermittent groups can be improved when given choice to intake all of their assigned energy in either one meal per day or divide it into small meals or snacks in a day, as long‐term adherence is considered more important than short‐term positive results from unrealistic or infeasible protocol. The conclusion of this discussion is in line with the systematic review by Cioffi et al. (2018) which states that the number of participants who were willing to continue IER beyond 6 months was lower than the calorie restriction group; therefore, it cannot be deduced that IER can be more easily followed than the CR.

Moreover, in the current review, TRE (16:8) was associated with significant reductions in body weight compared to the control group (Shafiee et al. 2025; Steger et al. 2023; Schroder et al. 2021; Kotarsky et al. 2021). Domaszewski et al. (2023) reported significant decrease in the weight in TRE 16:8 group compared to the control group following normal dietary pattern but receiving an educational program. Adherence percentage of 99% in females and 98% in males, with age range of 64–75 years indicates that 16:8 intermittent fasting can be easily followed by older adults for short term. These findings are consistent with other trials reflecting their effectiveness in weight related outcomes. For instance, two studies (Chow et al. 2020; Gabel et al. 2018) compared TRE (16:8) with a control group following a usual diet over 12 weeks. Gabel et al. (2018) reported approximately 3% greater weight reduction in the TRE group, while Chow et al. (2020) observed a 3.7% decrease in body weight in the TRE group compared to the control. A weight loss of 5%–10% of baseline weight is sufficient to yield cardiovascular health benefits (Brown et al. 2016). The results of a study by Lowe et al. (2020) are contrary to those stated before for TRE 16:8. The study stated that the weight loss results of TRE 16:8 alone without any energy restriction during feast days and any structured exercise, did not differ significantly from Consistent Meal Timing (CME) control group which contained three structured meals throughout the day, when followed for 12 weeks. Similarly, Rizvi et al. (2024) did not indicate any significant differences in the BMI of three groups or in other words, the weight did not variate significantly among the three groups, that is, Group A (16:8 IF), Group B (customized diet plan or CD group) and Group C (regular diet). An important study by Martínez‐Montoro et al. (2025) found no significant difference in weight loss between the 16:8 eTRE group and MedDiet control group while other groups such as Ketogenic diet (KD), lTRE and mADF found significant differences from the MedDiet control group.

Apparently, exercise combined with intermittent fasting can be an important strategy to lose weight as proven in the study of Bhutani et al. (2013) which states that ADF when combined with moderate intensity exercise reduces more weight than any of the one intervention if practiced alone. This result is in line with the findings of a meta‐analysis by Kazeminasab et al. (2024) which found that intermittent fasting practiced with physical activity is better than control combined with exercise resulting in better weight loss outcomes. This result is contradicted in the study by Ezpeleta et al. (2023) which shows that weight loss results of ADF when combined with moderate intensity aerobic exercise are comparable with ADF alone, but better than the exercise‐only group. Another study by Fadhadli et al. (2021) contradicts that hypothesis and states that 16:8 TRF alone and 16:8 TRF with brisk walk/BW (1 h in a day, 5 days a week) did not produce significantly different results in weight loss.

One study indicated that the 16:8 TRE regimen was more effective at reducing triglycerides than ADF (Chair et al. 2022). However, three cases of dizziness were reported in the TRE group, with none in the ADF group, suggesting that ADF is safer. The same study stated an important result that 16:8 TRF is less effective in reducing weight as compared to ADF during the 3 weeks of intervention thus proving that ADF is better in terms of adherence and end results. This study is important as few studies compare IF types with each other. However, it is important to consider that no clinically dangerous laboratory abnormalities have been observed in the TRE group (Anic et al. 2022). Eshghinia and Mohammadzadeh (2013) demonstrated that an 8‐week ADF intervention resulted in significant weight loss (*p* < 0.001), supporting its efficacy and safety in the short term.

A study by Cienfuegos et al. (2020) conducted for 10 weeks that explored the weight loss effects of 4 h (ad libitum intake during 4 h eating window) and 6 h TRF (ad libitum intake during 6 h eating window) in comparison with control having no time restrictions for meals. The eating window consisted of ad libitum intake of food without any calorie restriction. The results indicated that there was no significant difference in the weight loss effects of 4 and 6 h TRF; however, both groups lost significant weight compared to the control. The study found that just limiting the time of eating window in a day has beneficial effects on the weight (Tsitsou et al. 2025; Quist et al. 2024; Talebi et al. 2024). Different studies have involved the assessment of effect of 14:10 TRE on weight loss outcomes (Tsitsou et al. 2025; Quist et al. 2024; Talebi et al. 2024) did not find any statistically significant difference between 14:10 TRE (without calorie restriction) and control group on a 3 month follow up post intervention. When 14:10 eTRE was followed with probiotic supplementation, it did not result in any significant difference from the 14:10 eTRE with placebo or DCR control group. Similarly, Tsitsou et al. (2025) measured the effect of early and late 14:10 TRF on weight loss and found no significant difference between the groups and the control. These results are in contrast with the study of Irani et al. (2024), which shows that participants in 14:10 TRE lose significantly more weight than control when both following calorie restriction.

### 4.2 Body Composition Outcomes

Changes in body composition, including fat mass, lean mass, body fat percentage, and waist circumference, also significantly impact overall health. Weight loss interventions have positive effects, including a decrease in fat mass, an improvement in lean mass, and a reduction in waist circumference. The results of the included studies have pointed out the impact of intermittent fasting protocols on different body composition markers. Both alternate day fasting (ADF) and time restricted feeding TRF (16:8) demonstrated body composition changes, that is, reductions in BMI and waist circumference in a shorter time period (3 weeks) (Chair et al. 2022). Similarly, different other intermittent fasting strategies such as IF (5:2) also documented significant BMI reductions in comparison with the control group, suggesting that these findings are not specific to a particular dietary protocol. The results are supported by a previous trial indicating the reductions in body weight and BMI after following IF (5:2) diet (Holmer et al. 2021). However, BMI alone does not clearly indicate the body composition changes as it fails to distinguish between fat and fat free mass. Therefore, its interpretation relies on body fat markers.

Numerous studies when compared intermittent fasting regimens (16:8 and 14:10) with control, found reduction in fat mass among the intermittent fasting treatments (Kotarsky et al. 2021; Kunduraci and Ozbek 2020). These findings are comparable to those of a previous 10‐week study in which implementation of Alternate Day Fasting (ADF), in combination with dietary counseling, reported significant fat mass reductions (Varady et al. 2009). Our results are consistent with other systematic reviews and meta‐analyses that have previously shown IF contributing to fat mass loss across different populations (Ashtary‐Larky et al. 2021; Khalafi et al. 2025). But an earlier analysis reported variability in fat mass loss, which could be attributed to intervention duration, protocol and adherence (Cho et al. 2019). Overall, the loss of adipose tissue favors the efficacy of these intermittent fasting strategies in the context of body composition markers.

Furthermore, changes in trunk fat offer a more detailed picture of the impact of intermittent fasting on body fat distribution, in addition to changes in fat mass. Trunk fat represents the central fat depots, which is more closely associated with metabolic dysfunction. Reductions in total and trunk fat in the TRE group compared to the control group may indicate the decrease in central obesity among the included studies (Steger et al. 2023; Lowe et al. 2020). These findings were supported by a previous study in which time restricted eating is found to be associated with decreasing abdominal fat as well as improving metabolic markers (Wilkinson et al. 2020). Changes in waist circumference further strengthens this pattern, as it is a reliable indicator of abdominal and subcutaneous fat, which is associated with an increased risk of non‐communicable diseases, such as diabetes and cardiovascular disease (Anuradha and Hemachandran 2012). Multiple studies demonstrated consistent waist circumference reductions in response of time restricting eating interventions, to control group (Shafiee et al. 2025; Rizvi et al. 2024).

Beyond waist circumference, different intermittent fasting protocols (TRE (16:8), ADF, 5:2 and Mediterranean based IF interventions) depicted no changes in fat‐free mass, compared to control or baseline (Oldenburg et al. 2025; Martínez‐Montoro et al. 2025; Ezpeleta et al. 2023; Steger et al. 2023; Lin et al. 2023; Witjaksono et al. 2022; Lowe et al. 2020). This demonstrates that intermittent fasting when followed without excessive calorie restriction doesn't compromise lean mass. But a few studies reported decreases in lean or muscle mass under certain circumstances. One study found a decline in fat‐free mass after a 14:10 time‐restricted eating protocol and another study found a transient decrease in muscle mass after 12 weeks of early and late time‐restricted feeding, but not at follow‐up (Quist et al. 2024; Tsitsou et al. 2025). These results suggest that lean mass may be affected by duration, feeding times and energy status.

Exercise itself imparts several benefits to an individual irrespective of calories restriction or intermittent fasting. Different studies found that intermittent fasting intervention when combined with exercise, led to muscle mass preservation, indicating the role of exercise in maintaining fat free mass during calorie restriction (Ezpeleta et al. 2023; Bhutani et al. 2013). In the context of previous literature, resistance‐trained males using TRF (16:8) had greater fat mass reduction with no change in fat‐free mass (Moro et al. 2016). Therefore, the combined intervention of intermittent fasting and exercise can significantly improve overall health indices, preserving lean mass and improving resting metabolic rate (Berk et al. 2021). All these findings collectively support the need for further long‐term studies to identify optimal IF protocols for different populations. This is indicated by Gu et al. (2022), who stated that the effects of intermittent fasting may differently appear in men and women, thus gender variability may occur. Given IF's emerging role in weight management, more comprehensive trials are needed to assess potential side effects or long‐term health benefits, including its possible impact on chronic disease prevention.

### 4.3 Long‐Term Weight Outcomes

Limited data is available on the long‐term impact of intermittent fasting (IF) on weight loss based on the studies included in this systematic review. Four studies were conducted with follow‐up phases. While in two studies, the duration of the trial was divided into weight loss and maintenance phases. A study comparing alternate day fasting (ADF) and time restricted feeding (TRF) found that ADF group lost more weight than the TRE group at the end of the follow‐up period. However, greater reductions in waist circumference were observed in the TRF group compared to ADF, but the reductions were not statistically significant (Chair et al. 2022). These results demonstrate that intermittent fasting approaches exert variable effects on different anthropometric markers. An included study demonstrated no differences between the intervention (TRE) and control groups from post‐intervention to the follow‐up period (Quist et al. 2024). Likewise, comparing intermittent calorie restriction with continuous calorie restriction also found no significant difference in long‐term weight maintenance suggesting that both strategies may have equivalent effects once the intervention has stopped (Schübel et al. 2018). In line with these findings, Tsitsou et al. (2025) found no differences between groups from the end of intervention to follow‐up, despite finding significant improvements from baseline to follow‐up in all groups. This finding that intermittent fasting is as effective as continuous calorie restriction is contrary to the results of a recent systematic review, which states that intermittent fasting is better than calorie restriction. Intermittent fasting does not decrease resting metabolic rate and consequently does not cause negative metabolic adaptation. It further states that intermittent fasting increases the energy required by the body to adapt to the irregular energy intake; therefore, it has the potential to enhance calorie consumption and proves better than continuous calorie restriction for weight loss (Zhang et al. 2025).

It is also worth noting that these follow‐up results should not be confused with the results of structured weight maintenance phases in some studies. Although these phases indicate weight maintenance with ongoing dietary counseling, they do not represent post‐intervention outcomes and may overestimate the long‐term efficacy of intermittent fasting. However, these studies suggest that adhering to intermittent fasting interventions might help in mitigating weight regain. These findings underscore the need for future studies with longer durations to comprehensively evaluate the long‐term sustainability and efficacy of various intermittent fasting protocols on weight and related health outcomes.

### 4.4 Mechanisms Underlying Weight Reduction and Other Health Effects Inferred by Intermittent Fasting

There are multiple physiological mechanisms through which intermittent fasting (IF) or time‐restricted eating (TRE) induces weight loss. A murine study demonstrated increased lipid utilization over carbohydrates under intermittent fasting, promoting elevated energy expenditure in every‐other‐day fasting (EODF) mice and inducing the beiging or browning of white adipose tissue (WAT) (Li et al. 2017). Findings from another study suggest that IF may promote weight loss even when periods of fasting are followed by compensatory overeating, provided there is no net increase in caloric intake, thus enhancing health and cellular resistance to disease (Anson et al. 2003). Another proposed mechanism for weight loss in intermittent fasting is its alignment with circadian rhythms, which further improves glucose metabolism, hormonal regulation, and sleep quality (Abdollahpour et al. 2025). Intermittent fasting also increases fatty acid oxidation, thereby decreasing hepatic triglyceride levels, which in turn leads to an enhancement of lipid profiles (Santos et al. 2022). Additionally, it has been demonstrated that mice receiving intermittent fasting had nearly twice the serum levels of ketones compared to those on continuous calorie restriction (Aksungar et al. 2017). These ketone bodies are further converted into protein molecules such as PPAR‐γ, brain‐derived neurotropic factor (BDNF) and ADP ribosyl cyclase, which stimulate catabolic autophagy and improve mitochondrial function (Cubeles‐Juberias et al. 2023). Collectively, increased lipid utilization, alignment with circadian rhythms, high fatty acid oxidation and levels of ketones shift the switch to lipid‐based metabolism. This switch is driven by depletion of the glycogen stores and intermittent calorie restriction (Conn et al. 2024).

### 4.5 Future Directions

The research findings should focus on identifying the type of intermittent fasting (IF) protocol, specifically 16:8 or 5:2 protocols, that are more effective for long‐term weight loss, as well as on the possible physiological effects underlying each of these processes. This will help dietitians and health experts develop more personalized and thus effective diet plans for overweight and obese patients. Moreover, research assessing weight maintenance over a longer period after IF is necessary. These studies should utilize larger sample sizes and include control groups to facilitate easier comparative assessment, such as the effect of the intervention and the non‐intervention arms.

### 4.6 Strengths and Limitations

This systematic review has several strengths and limitations. The long period chosen (2000–2025) is a significant strength, as it provides a comprehensive picture of the impact of IF on weight loss, encompassing 25 years of data. The intervention involved all studies that used control groups, which enabled tracing the effects of the intervention and normal conditions more easily. The inclusion criteria were broad in terms of age range (18–75 years), thereby encompassing the representation of young, middle‐aged, and older adults. Further confounding factors, such as metformin drugs, were avoided, thereby isolating the actual effects of IF treatments. The PRISMA guidelines were used to review the methodology and ensure the review was of high quality, transparent, and reproducible. Above all, it considered weight‐related outcomes only to ensure the accuracy in assessing the effectiveness of IF protocols. In addition, risk of bias assessment (RoB2) revealed “low risk” for majority of the randomized controlled studies, which enhances the confidence in the observed effects.

However, some limitations must be acknowledged. The strict inclusion criteria reduced the number of eligible studies. The gender distribution was imbalanced, with approximately 71% female and 29% male participants, which may have skewed the results. Only English‐language articles were reviewed, which may have excluded relevant research. Differences in how weight‐related outcomes were reported, some using kilograms and others percentages, made inter‐study comparisons more difficult. Heterogeneity in study design, intervention duration, and fasting protocols prevented the possibility of meta‐analysis.

## 5 Conclusion

The prevalence of overweight and obesity is increasing day by day. To overcome this, different dietary strategies have been recommended. This systematic review shows that IF can be a promising dietary approach for weight loss to treat overweight and obesity in different populations including young adults, diseased populations, and exercise combined interventions, with no better results than continuous calorie restriction (CCR). The results show that IF not only causes reductions in body weight but also improves body composition measures, such as fat mass, lean mass, percentage body fat, and waist circumference. However, these findings cannot be generalized to other populations of overweight and obese people including teens (less than 18 years), elderly (above 75 years), and comorbid adults, because these populations are not under the scope of this review, warranting caution while interpreting them. Moreover, the confidence in these findings is influenced by moderate bias risk. Further high‐quality studies with a longer duration are needed to investigate its long‐term outcomes and to understand its role in achieving sustainable weight loss.

## Author Contributions

**Hafiz Muhammad Irfan Manzoor:** conceptualization. **Ahmad Mujtaba Noman:** writing – original draft. **Aiman Sohail:** writing – original draft. **Muhammad Tauseef Sultan:** conceptualization, writing – original draft. **Maheen Kazmi:** writing – review and editing. **Mohamed Fawzy Ramadan:** funding acquisition, writing – review and editing. **Mahnoor Khalid:** writing – review and editing. **Fahad Al‐Asmari:** validation, funding acquisition, writing – review and editing. **Eliasse Zongo:** funding acquisition, writing – review and editing. **Muhammad Abdul Rahim:** writing – review and editing. **Aneela Hameed:** conceptualization.

## Funding

This work was supported by the Deanship of Scientific Research, Vice Presidency for Graduate Studies and Scientific Research, King Faisal University, Saudi Arabia (Grant No. KFU264280).

## Ethics Statement

The authors have nothing to report.

## Consent

The authors have nothing to report.

## Conflicts of Interest

The authors declare no conflicts of interest.

## Supporting information


**Appendix A.** fsn372264‐sup‐0001‐AppendixA.xlsm.

**Appendix B.** fsn372264‐sup‐0002‐AppendixB.xlsx.

## Data Availability

The data that support the findings of this study are available on request from the corresponding author.

## References

[fsn372264-bib-0001] Abdollahpour, N. , N. Seifi , M. Nosrati , et al. 2025. “Intermittent Fasting and Body Composition: Insights From the Iranian National Obesity Registry (IRNOR).” Human Nutrition & Metabolism 41: 200316. 10.1016/j.hnm.2025.200316.

[fsn372264-bib-0002] Ahmed, N. , J. Farooq , H. S. Siddiqi , et al. 2021. “Impact of Intermittent Fasting on Lipid Profile‐A Quasi‐Randomized Clinical Trial.” Frontiers in Nutrition 7: 96787. 10.3389/fnut.2020.596787. PMC7882512 33598473

[fsn372264-bib-0004] Aksungar, F. B. , M. Sarıkaya , A. Coskun , M. Serteser , and I. Unsal . 2017. “Comparison of Intermittent Fasting Versus Caloric Restriction in Obese Subjects: A Two Year Follow‐Up.” Journal of Nutrition, Health & Aging 21, no. 6: 681–685. 10.1007/s12603-016-0786-y. PMC12879802 28537332

[fsn372264-bib-0005] Anic, K. , M. W. Schmidt , L. Furtado , et al. 2022. “Intermittent Fasting‐Short‐ and Long‐Term Quality of Life, Fatigue, and Safety in Healthy Volunteers: A Prospective, Clinical Trial.” Nutrients 14, no. 19: 4216. 10.3390/nu14194216. 36235868 PMC9571750

[fsn372264-bib-0006] Anson, R. M. , Z. Guo , R. de Cabo , et al. 2003. “Intermittent Fasting Dissociates Beneficial Effects of Dietary Restriction on Glucose Metabolism and Neuronal Resistance to Injury From Calorie Intake.” Proceedings of the National Academy of Sciences of the United States of America 100, no. 10: 6216–6220. 10.1073/pnas.1035720100. 12724520 PMC156352

[fsn372264-bib-0007] Anuradha, R. , and S. Hemachandran . 2012. “The Waist Circumference Measurement: A Simple Method for Assessing the Abdominal Obesity.” Journal of Clinical and Diagnostic Research: JCDR 6, no. 9: 1510–1513. 10.7860/JCDR/2012/4379.2545. 23285442 PMC3527782

[fsn372264-bib-0008] Ashtary‐Larky, D. , R. Bagheri , G. M. Tinsley , O. Asbaghi , A. Paoli , and T. Moro . 2021. “Effects of Intermittent Fasting Combined With Resistance Training on Body Composition: A Systematic Review and Meta‐Analysis.” Physiology & Behavior 237: 113453. 10.1016/j.physbeh.2021.113453. 33984329

[fsn372264-bib-0009] Beaulieu, K. , N. Casanova , P. Oustric , et al. 2020. “Matched Weight Loss Through Intermittent or Continuous Energy Restriction Does Not Lead to Compensatory Increases in Appetite and Eating Behavior in a Randomized Controlled Trial in Women With Overweight and Obesity.” Journal of Nutrition 150, no. 3: 623–633. 10.1093/jn/nxz296. 31825067

[fsn372264-bib-0010] Berk, Y. , Ş. Ünver , and H. Avlayan . 2021. “The Effect of Intermittent Fasting and Exercise on Some Physiological Parameters.” Pakistan Journal of Medical and Health Sciences 15, no. 9: 2793–2798. 10.53350/pjmhs211592793.

[fsn372264-bib-0011] Bhutani, S. , M. C. Klempel , C. M. Kroeger , J. F. Trepanowski , and K. A. Varady . 2013. “Alternate Day Fasting and Endurance Exercise Combine to Reduce Body Weight and Favorably Alter Plasma Lipids in Obese Humans.” Obesity (Silver Spring, Md) 21, no. 7: 1370–1379. 10.1002/oby.20353. 23408502

[fsn372264-bib-0012] Brown, J. D. , J. Buscemi , V. Milsom , R. Malcolm , and P. M. O'Neil . 2016. “Effects on Cardiovascular Risk Factors of Weight Losses Limited to 5–10.” Translational Behavioral Medicine 6, no. 3: 339–346. 10.1007/s13142-015-0353-9. 27528523 PMC4987606

[fsn372264-bib-0013] Catenacci, V. A. , Z. Pan , D. Ostendorf , et al. 2016. “A Randomized Pilot Study Comparing Zero‐Calorie Alternate‐Day Fasting to Daily Caloric Restriction in Adults With Obesity.” Obesity (Silver Spring, Md) 24, no. 9: 1874–1883. 10.1002/oby.21581. 27569118 PMC5042570

[fsn372264-bib-0014] Chair, S. Y. , H. Cai , X. Cao , Y. Qin , H. Y. Cheng , and M. T. Ng . 2022. “Intermittent Fasting in Weight Loss and Cardiometabolic Risk Reduction: A Randomized Controlled Trial.” Journal of Nursing Research: JNR 30, no. 1: e185. 10.1097/jnr.0000000000000469. 35050952

[fsn372264-bib-0016] Cho, Y. , N. Hong , K. W. Kim , et al. 2019. “The Effectiveness of Intermittent Fasting to Reduce Body Mass Index and Glucose Metabolism: A Systematic Review and Meta‐Analysis.” Journal of Clinical Medicine 8, no. 10: 1645. 10.3390/JCM8101645. 31601019 PMC6832593

[fsn372264-bib-0017] Chow, L. S. , E. N. C. Manoogian , A. Alvear , et al. 2020. “Time‐Restricted Eating Effects on Body Composition and Metabolic Measures in Humans Who Are Overweight: A Feasibility Study.” Obesity (Silver Spring, Md) 28, no. 5: 860–869. 10.1002/oby.22756. PMC7180107 32270927

[fsn372264-bib-0018] Cienfuegos, S. , K. Gabel , F. Kalam , et al. 2020. “Effects of 4‐ and 6‐h Time‐Restricted Feeding on Weight and Cardiometabolic Health: A Randomized Controlled Trial in Adults With Obesity.” Cell Metabolism 32, no. 3: 366–378.e3. 10.1016/j.cmet.2020.06.018. 32673591 PMC9407646

[fsn372264-bib-0019] Cioffi, I. , A. Evangelista , V. Ponzo , et al. 2018. “Intermittent Versus Continuous Energy Restriction on Weight Loss and Cardiometabolic Outcomes: A Systematic Review and Meta‐Analysis of Randomized Controlled Trials.” Journal of Translational Medicine 16, no. 1: 371. 10.1186/S12967-018-1748-4. 30583725 PMC6304782

[fsn372264-bib-0020] Conn, M. O. , D. M. Marko , and J. D. Schertzer . 2024. “Intermittent Fasting Increases Fat Oxidation and Promotes Metabolic Flexibility in Lean Mice but Not Obese Type 2 Diabetic Mice.” American Journal of Physiology. Endocrinology and Metabolism 327, no. 4: E470–E477. 10.1152/ajpendo.00255.2024. 39196802

[fsn372264-bib-0078] Cubeles‐Juberias, E. , L. Herrero , and M. d. M. Romero . 2023. “Effects of Intermittent Fasting on Metabolism, Cognitive Function, and Aging: A Review of Human and Animal Studies.” Journal of the Spanish Society of Obesity and Metabolic Surgery and of the Spanish Society for the Study of Obesity 13, no. 3: 4197–4202. 10.53435/funj.00924.

[fsn372264-bib-0023] Diab, R. , L. Dimachkie , O. Zein , A. Dakroub , and A. H. Eid . 2024. “Intermittent Fasting Regulates Metabolic Homeostasis and Improves Cardiovascular Health.” Cell Biochemistry and Biophysics 82, no. 3: 1583–1597. 10.1007/s12013-024-01314-9. 38847940 PMC11445340

[fsn372264-bib-0075] Domaszewski, P. , M. Konieczny , T. Dybek , et al. 2023. “Comparison of the Effects of Six‐Week Time‐Restricted Eating on Weight Loss, Body Composition, and Visceral Fat in Overweight Older Men and Women.” Experimental Gerontology 174: 112116. 10.1016/j.exger.2023.112116. 36739795

[fsn372264-bib-0024] Erdem, N. Z. , E. Bayraktaroğlu , R. A. Samancı , E. Geçgil‐Demir , N. G. Tarakçı , and F. Mert‐Biberoğlu . 2022. “The Effect of Intermittent Fasting Diets on Body Weight and Composition.” Clinical Nutrition ESPEN 51: 207–214. 10.1016/j.clnesp.2022.08.030. 36184206

[fsn372264-bib-0025] Eshghinia, S. , and F. Mohammadzadeh . 2013. “The Effects of Modified Alternate‐Day Fasting Diet on Weight Loss and CAD Risk Factors in Overweight and Obese Women.” Journal of Diabetes and Metabolic Disorders 12, no. 1: 4. 10.1186/2251-6581-12-4. 23497604 PMC3598220

[fsn372264-bib-0026] Ezpeleta, M. , K. Gabel , S. Cienfuegos , et al. 2023. “Effect of Alternate Day Fasting Combined With Aerobic Exercise on Non‐Alcoholic Fatty Liver Disease: A Randomized Controlled Trial.” Cell Metabolism 35, no. 1: 56–70.e3. 10.1016/j.cmet.2022.12.001. 36549296 PMC9812925

[fsn372264-bib-0027] Fadhadli, F. , S. Aung , N. Noor , and A. Razali . 2021. “Time‐Restricted Feeding and Brisk Walking in Overweight and Obese Adults.” Malaysian Journal of Medicine and Health Sciences 17, no. 4: 95–101.

[fsn372264-bib-0028] Gabel, K. , K. K. Hoddy , N. Haggerty , et al. 2018. “Effects of 8‐Hour Time Restricted Feeding on Body Weight and Metabolic Disease Risk Factors in Obese Adults: A Pilot Study.” Nutrition and Healthy Aging 4, no. 4: 345–353. 10.3233/NHA-170036. 29951594 PMC6004924

[fsn372264-bib-0029] Gu, L. , R. Fu , J. Hong , H. Ni , K. Yu , and H. Lou . 2022. “Effects of Intermittent Fasting in Human Compared to a Non‐Intervention Diet and Caloric Restriction: A Meta‐Analysis of Randomized Controlled Trials.” Frontiers in Nutrition 9: 871682. 10.3389/fnut.2022.871682. 35586738 PMC9108547

[fsn372264-bib-0030] Harris, L. , S. Hamilton , L. B. Azevedo , et al. 2018. “Intermittent Fasting Interventions for Treatment of Overweight and Obesity in Adults: A Systematic Review and Meta‐Analysis.” JBI Database of Systematic Reviews and Implementation Reports 16, no. 2: 507–547. 10.11124/JBISRIR-2016-003248. 29419624

[fsn372264-bib-0031] Headland, M. L. , P. M. Clifton , and J. B. Keogh . 2019. “Effect of Intermittent Compared to Continuous Energy Restriction on Weight Loss and Weight Maintenance After 12 Months in Healthy Overweight or Obese Adults.” International Journal of Obesity 43, no. 10: 2028–2036. 10.1038/s41366-018-0247-2. 30470804

[fsn372264-bib-0032] Holmer, M. , C. Lindqvist , S. Petersson , et al. 2021. “Treatment of NAFLD With Intermittent Calorie Restriction or Low‐Carb High‐Fat Diet—A Randomised Controlled Trial.” JHEP Reports 3, no. 3: 100256. 10.1016/j.jhepr.2021.100256. 33898960 PMC8059083

[fsn372264-bib-0033] Hong, Q. N. , P. Pluye , S. Fàbregues , et al. 2018. “Mixed Methods Appraisal Tool (MMAT) Version 2018 User Guide.” http://mixedmethodsappraisaltoolpublic.pbworks.com/.

[fsn372264-bib-0034] Hooshiar, S. H. , A. Yazdani , and S. Jafarnejad . 2023. “Alternate‐Day Modified Fasting Diet Improves Weight Loss, Subjective Sleep Quality and Daytime Dysfunction in Women With Obesity or Overweight: A Randomized, Controlled Trial.” Frontiers in Nutrition 10: 1174293. 10.3389/FNUT.2023.1174293. 37275639 PMC10233006

[fsn372264-bib-0035] Hutchison, A. T. , B. Liu , R. E. Wood , et al. 2019. “Effects of Intermittent Versus Continuous Energy Intakes on Insulin Sensitivity and Metabolic Risk in Women With Overweight.” Obesity (Silver Spring, Md) 27, no. 1: 50–58. 10.1002/OBY.22345. 30569640

[fsn372264-bib-0079] Irani, H. , B. Abiri , B. Khodami , et al. 2024. “Effect of Time Restricted Feeding on Anthropometric Measures, Eating Behavior, Stress, Serum Levels of BDNF and LBP in Overweight/Obese Women With Food Addiction: A Randomized Clinical Trial.” Nutritional Neuroscience 27, no. 6: 577–589. 10.1080/1028415X.2023.2234704. 37436939

[fsn372264-bib-0080] Kazeminasab, F. , M. Baharlooie , B. Karimi , K. Mokhtari , S. K. Rosenkranz , and H. O. Santos . 2024. “Effects of Intermittent Fasting Combined With Physical Exercise on Cardiometabolic Outcomes: Systematic Review and Meta‐Analysis of Clinical Studies.” Nutrition Reviews 82, no. 12: 1726–1740. 10.1093/NUTRIT/NUAD155. 38102800

[fsn372264-bib-0038] Khalafi, M. , A. H. Maleki , M. Ehsanifar , M. E. Symonds , and S. K. Rosenkranz . 2025. “Longer‐Term Effects of Intermittent Fasting on Body Composition and Cardiometabolic Health in Adults With Overweight and Obesity: A Systematic Review and Meta‐Analysis.” Obesity Reviews 26, no. 2: e13855. 10.1111/obr.13855. 39501676

[fsn372264-bib-0039] Klempel, M. C. , C. M. Kroeger , S. Bhutani , J. F. Trepanowski , and K. A. Varady . 2012. “Intermittent Fasting Combined With Calorie Restriction Is Effective for Weight Loss and Cardio‐Protection in Obese Women.” Nutrition Journal 11: 98. 10.1186/1475-2891-11-98. 23171320 PMC3511220

[fsn372264-bib-0040] Kord Varkaneh, H. , A. Salehi sahlabadi , M. A. Găman , et al. 2022. “Effects of the 5:2 Intermittent Fasting Diet on Non‐Alcoholic Fatty Liver Disease: A Randomized Controlled Trial.” Frontiers in Nutrition 9: 948655. 10.3389/fnut.2022.948655. 35958257 PMC9360602

[fsn372264-bib-0077] Kotarsky, C. J. , N. R. Johnson , S. J. Mahoney , et al. 2021. “Time‐Restricted Eating and Concurrent Exercise Training Reduces Fat Mass and Increases Lean Mass in Overweight and Obese Adults.” Physiological Reports 9, no. 10: e14868. 10.14814/phy2.14868. 34042299 PMC8157764

[fsn372264-bib-0041] Kunduraci, Y. E. , and H. Ozbek . 2020. “Does the Energy Restriction Intermittent Fasting Diet Alleviate Metabolic Syndrome Biomarkers? A Randomized Controlled Trial.” Nutrients 12, no. 10: 1–13. 10.3390/NU12103213. PMC7589692 33096684

[fsn372264-bib-0042] Li, G. , C. Xie , S. Lu , et al. 2017. “Intermittent Fasting Promotes White Adipose Browning and Decreases Obesity by Shaping the Gut Microbiota.” Cell Metabolism 26, no. 4: 672–685.e4. 10.1016/j.cmet.2017.08.019. 28918936 PMC5668683

[fsn372264-bib-0043] Lin, S. , S. Cienfuegos , M. Ezpeleta , et al. 2023. “Effect of Time‐Restricted Eating Versus Daily Calorie Restriction on Mood and Quality of Life in Adults With Obesity.” Nutrients 15, no. 20: 4313. 10.3390/nu15204313. 37892388 PMC10609268

[fsn372264-bib-0044] Lowe, D. A. , N. Wu , L. Rohdin‐Bibby , et al. 2020. “Effects of Time‐Restricted Eating on Weight Loss and Other Metabolic Parameters in Women and Men With Overweight and Obesity: The TREAT Randomized Clinical Trial.” JAMA Internal Medicine 180, no. 11: 1491–1499. 10.1001/jamainternmed.2020.4153. 32986097 PMC7522780

[fsn372264-bib-0045] Martínez‐Montoro, J. I. , B. Bandera , M. Gutiérrez‐Bedmar , et al. 2025. “Effect of a Ketogenic Diet, Time‐Restricted Eating, or Alternate‐Day Fasting on Weight Loss in Adults With Obesity: A Randomized Clinical Trial.” BMC Medicine 23, no. 1: 368. 10.1186/S12916-025-04182-Z. 40598397 PMC12220164

[fsn372264-bib-0046] Martínez‐Rodríguez, A. , J. A. Rubio‐Arias , J. M. García‐De Frutos , M. Vicente‐Martínez , and T. P. Gunnarsson . 2021. “Effect of High‐Intensity Interval Training and Intermittent Fasting on Body Composition and Physical Performance in Active Women.” International Journal of Environmental Research and Public Health 18, no. 12: 6431. 10.3390/ijerph18126431. 34198554 PMC8296247

[fsn372264-bib-0048] Moro, T. , G. Tinsley , A. Bianco , et al. 2016. “Effects of Eight Weeks of Time‐Restricted Feeding (16/8) on Basal Metabolism, Maximal Strength, Body Composition, Inflammation, and Cardiovascular Risk Factors in Resistance‐Trained Males.” Journal of Translational Medicine 14, no. 1: 290. 10.1186/s12967-016-1044-0. 27737674 PMC5064803

[fsn372264-bib-0049] Oldenburg, N. , D. G. Mashek , L. Harnack , et al. 2025. “Time‐Restricted Eating, Caloric Reduction, and Unrestricted Eating Effects on Weight and Metabolism: A Randomized Trial.” Obesity (Silver Spring, Md) 33, no. 4: 671–684. 10.1002/oby.24252. 39973006 PMC11937878

[fsn372264-bib-0051] Patterson, R. E. , and D. D. Sears . 2017. “Metabolic Effects of Intermittent Fasting.” Annual Review of Nutrition 37: 371–393. 10.1146/annurev-nutr-071816-064634. PMC13170603 28715993

[fsn372264-bib-0074] Quist, J. S. , H. E. Pedersen , M. M. Jensen , et al. 2024. “Effects of 3 Months of 10‐h Per‐Day Time‐Restricted Eating and 3 Months of Follow‐Up on Bodyweight and Cardiometabolic Health in Danish Individuals at High Risk of Type 2 Diabetes: The RESET Single‐Centre, Parallel, Superiority, Open‐Label, Randomised Controlled Trial.” Lancet. Healthy Longevity 5, no. 5: e314–e325. 10.1016/S2666-7568(24)00028-X. 38588687

[fsn372264-bib-0052] Rizvi, Z. A. , J. Saleem , I. Zeb , et al. 2024. “Effects of Intermittent Fasting on Body Composition, Clinical Health Markers and Memory Status in the Adult Population: A Single‐Blind Randomised Controlled Trial.” Nutrition Journal 23, no. 1: 147. 10.1186/S12937-024-01046-9. 39609683 PMC11603954

[fsn372264-bib-0053] Salis, S. , S. Shefa , N. Sharma , et al. 2022. “Effects of Intermittent Fasting on Weight Loss in Asian Indian Adults With Obesity.” Journal of the Association of Physicians of India 70, no. 9: 11–12. 10.5005/japi-11001-0098. 36082888

[fsn372264-bib-0054] Santos, H. O. , R. Genario , G. M. Tinsley , et al. 2022. “A Scoping Review of Intermittent Fasting, Chronobiology, and Metabolism.” American Journal of Clinical Nutrition 115, no. 4: 991–1004. 10.1093/ajcn/nqab433. 34978321

[fsn372264-bib-0055] Schroder, J. D. , H. Falqueto , A. Mânica , et al. 2021. “Effects of Time‐Restricted Feeding in Weight Loss, Metabolic Syndrome and Cardiovascular Risk in Obese Women.” Journal of Translational Medicine 19, no. 1: 3. 10.1186/s12967-020-02687-0. 33407612 PMC7786967

[fsn372264-bib-0056] Schübel, R. , J. Nattenmüller , D. Sookthai , et al. 2018. “Effects of Intermittent and Continuous Calorie Restriction on Body Weight and Metabolism Over 50 Wk: A Randomized Controlled Trial.” American Journal of Clinical Nutrition 108, no. 5: 933–945. 10.1093/AJCN/NQY196. 30475957 PMC6915821

[fsn372264-bib-0057] Semnani‐Azad, Z. , T. A. Khan , L. Chiavaroli , et al. 2025. “Intermittent Fasting Strategies and Their Effects on Body Weight and Other Cardiometabolic Risk Factors: Systematic Review and Network Meta‐Analysis of Randomised Clinical Trials.” BMJ (Clinical Research ed.) 389: e082007. 10.1136/bmj-2024-082007. PMC12175170 40533200

[fsn372264-bib-0073] Shafiee, M. , A. Sadeghi , F. Ghafouri‐Taleghani , et al. 2025. “Effects of Time Restricted Feeding Combined With Lacto Ovo Vegetarian Diet on Metabolic Associated Fatty Liver Disease Management: A Randomized Clinical Trial.” Scientific Reports 15, no. 1: 4463. 10.1038/s41598-025-88773-z. 39915600 PMC11803106

[fsn372264-bib-0058] Song, D. K. , and Y. W. Kim . 2023. “Beneficial Effects of Intermittent Fasting: A Narrative Review.” Journal of Yeungnam Medical Science 40, no. 1: 4–11. 10.12701/jyms.2022.00010. 35368155 PMC9946909

[fsn372264-bib-0059] Steger, F. L. , H. Jamshed , D. R. Bryan , et al. 2023. “Early Time‐Restricted Eating Affects Weight, Metabolic Health, Mood, and Sleep in Adherent Completers: A Secondary Analysis.” Obesity (Silver Spring, Md) 31, no. Suppl 1: 96–107. 10.1002/oby.23614. PMC9877132 36518092

[fsn372264-bib-0060] Sterne, J. A. C. , J. Savović , M. J. Page , et al. 2019. “RoB 2: A Revised Tool for Assessing Risk of Bias in Randomised Trials.” BMJ (Clinical Research ed.) 366: l4898. 10.1136/bmj.l4898. 31462531

[fsn372264-bib-0061] Sun, M. L. , W. Yao , X. Y. Wang , et al. 2024. “Intermittent Fasting and Health Outcomes: An Umbrella Review of Systematic Reviews and Meta‐Analyses of Randomised Controlled Trials.” EClinicalMedicine 70: 102519. 10.1016/j.eclinm.2024.102519. 38500840 PMC10945168

[fsn372264-bib-0062] Sundfør, T. M. , M. Svendsen , and S. Tonstad . 2018. “Effect of Intermittent Versus Continuous Energy Restriction on Weight Loss, Maintenance and Cardiometabolic Risk: A Randomized 1‐Year Trial.” Nutrition, Metabolism, and Cardiovascular Diseases 28, no. 7: 698–706. 10.1016/j.numecd.2018.03.009. 29778565

[fsn372264-bib-0063] Talebi, S. , S. Shab‐Bidar , A. Moini , H. Mohammadi , and K. Djafarian . 2024. “The Effects of Time‐Restricted Eating Alone or in Combination With Probiotic Supplementation in Comparison With a Calorie‐Restricted Diet on Endocrine and Metabolic Profiles in Women With Polycystic Ovary Syndrome: A Randomized Clinical Trial.” Diabetes, Obesity and Metabolism 26, no. 10: 4468–4479. 10.1111/DOM.15801. 39143654

[fsn372264-bib-0064] Trepanowski, J. F. , C. M. Kroeger , A. Barnosky , et al. 2017. “Effect of Alternate‐Day Fasting on Weight Loss, Weight Maintenance, and Cardioprotection Among Metabolically Healthy Obese Adults: A Randomized Clinical Trial.” JAMA Internal Medicine 177, no. 7: 930–938. 10.1001/jamainternmed.2017.0936. 28459931 PMC5680777

[fsn372264-bib-0065] Tsitsou, S. , T. Bali , M. Adamantou , et al. 2025. “Effects of a 12‐Week Mediterranean‐Type Time‐Restricted Feeding Protocol in Patients With Metabolic Dysfunction‐Associated Steatotic Liver Disease: A Randomised Controlled Trial—The ‘CHRONO‐NAFLD Project’.” Alimentary Pharmacology & Therapeutics 61, no. 8: 1290–1309. 10.1111/APT.70044. 40017349 PMC11950810

[fsn372264-bib-0066] Varady, K. A. , S. Bhutani , E. C. Church , and M. C. Klempel . 2009. “Short‐Term Modified Alternate‐Day Fasting: A Novel Dietary Strategy for Weight Loss and Cardioprotection in Obese Adults.” American Journal of Clinical Nutrition 90, no. 5: 1138–1143. 10.3945/ajcn.2009.28380. 19793855

[fsn372264-bib-0067] Varady, K. A. , S. Bhutani , M. C. Klempel , and C. M. Kroeger . 2011. “Comparison of Effects of Diet Versus Exercise Weight Loss Regimens on LDL and HDL Particle Size in Obese Adults.” Lipids in Health and Disease 10: 119. 10.1186/1476-511X-10-119. 21767400 PMC3150311

[fsn372264-bib-0068] Wilkinson, M. J. , E. N. Manoogian , A. Zadourian , et al. 2020. “Ten‐Hour Time‐Restricted Eating Reduces Weight, Blood Pressure, and Atherogenic Lipids in Patients With Metabolic Syndrome.” Cell Metabolism 31, no. 1: 92–104. 10.1016/j.cmet.2019.11.004. 31813824 PMC6953486

[fsn372264-bib-0069] Wilson, R. A. , W. Deasy , C. G. Stathis , A. Hayes , and M. B. Cooke . 2018. “Intermittent Fasting With or Without Exercise Prevents Weight Gain and Improves Lipids in Diet‐Induced Obese Mice.” Nutrients 10, no. 3: 346. 10.3390/nu10030346. 29534545 PMC5872764

[fsn372264-bib-0070] Witjaksono, F. , E. Prafiantini , and A. Rahmawati . 2022. “Effect of Intermittent Fasting 5:2 on Body Composition and Nutritional Intake Among Employees With Obesity in Jakarta: A Randomized Clinical Trial.” BMC Research Notes 15, no. 1: 323. 10.1186/S13104-022-06209-7. 36224641 PMC9559012

[fsn372264-bib-0071] World Health Organization . 2020. “Obesity and Overweight.” https://www.who.int/news‐room/fact‐sheets/detail/obesity‐and‐overweight.

[fsn372264-bib-0072] Zhang, S. , B. Sun , L. Sun , S. Zou , and Q. Chen . 2025. “Effect of Intermittent Fasting on Obesity and Metabolic Indices in Patients With Metabolic Syndrome: A Systematic Review and Meta Analysis.” BMC Endocrine Disorders 25, no. 1: 130. 10.1186/s12902-025-01952-x. 40369509 PMC12076832

